# Paternal heterochromatin formation in human embryos is H3K9/HP1 directed and primed by sperm-derived histone modifications

**DOI:** 10.1038/ncomms6868

**Published:** 2014-12-18

**Authors:** Christine van de Werken, Godfried W. van der Heijden, Cindy Eleveld, Miriam Teeuwssen, Mareike Albert, Willy M. Baarends, Joop S. E. Laven, Antoine H. F. M. Peters, Esther B. Baart

**Affiliations:** 1 Division of Reproductive Medicine, Department of Obstetrics and Gynaecology, Erasmus MC, University Medical Center, Postbus 2040, 3000 CA Rotterdam, The Netherlands; 2 Department of Reproduction and Development, Erasmus MC, University Medical Center, Postbus 2040, 3000 CA Rotterdam, The Netherlands; 3 Friedrich Miescher Institute for Biomedical Research, Maulbeerstrasse 66, 4058 Basel, Switzerland; 4 Faculty of Sciences, University of Basel, Klingelbergstrasse 50, 4056 Basel, Switzerland

## Abstract

The different configurations of maternal and paternal chromatin, acquired during oogenesis and spermatogenesis, have to be rearranged after fertilization to form a functional embryonic genome. In the paternal genome, nucleosomal chromatin domains are re-established after the protamine-to-histone exchange. We investigated the formation of constitutive heterochromatin (cHC) in human preimplantation embryos. Our results show that histones carrying canonical cHC modifications are retained in cHC regions of sperm chromatin. These modified histones are transmitted to the oocyte and contribute to the formation of paternal embryonic cHC. Subsequently, the modifications are recognized by the H3K9/HP1 pathway maternal chromatin modifiers and propagated over the embryonic cleavage divisions. These results are in contrast to what has been described for mouse embryos, in which paternal cHC lacks canonical modifications and is initially established by Polycomb group proteins. Our results show intergenerational epigenetic inheritance of the cHC structure in human embryos.

## Abstract (web summary)

Following fertilization, the oocyte and sperm lose their distinct chromatin signature to form a functional embryonic genome. Here the authors find that, in human embryos, the paternal constitutive heterochromatin is inherited in the canonical configuration from the sperm and is propagated by the H3K9/HP1 pathway.

## Graphical abstract

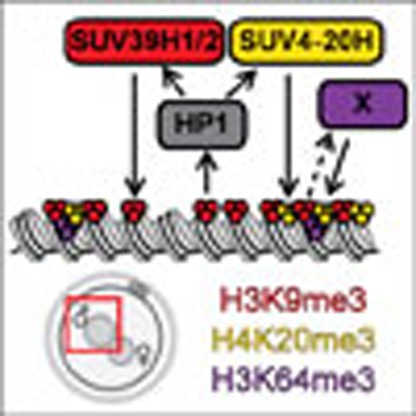

Fertilization marks the fusion of two specialized gametes—oocyte and sperm. In mammalian zygotes, the maternal and paternal genomes exist in an asymmetric chromatin configuration. Extensive reorganization of chromatin to the embryonic configuration is crucial for the developmental potency^1^. During this process, some information of parental origin needs to be retained to maintain imprinting^2^. Other chromatin domains, such as the constitutive heterochromatin (cHC), need to be reorganized to the somatic configuration to function properly^3^,^4^.

Constitutive HC assembles mostly on telomeric, centromeric and pericentric regions, remains condensed throughout the cell cycle and is important for genome stability and chromosome segregation^5^. DNA sequences underlying cHC differ between species, but mainly consist of repeats and transposons. In mouse, most of the cHC is located pericentrically (pericentric heterochromatin (pHC)), a region with major satellite DNA repeats. In human, cHC is more dispersed across the genome^6^; classic satellite II and III DNA repeats localize to the pericentric region, but also to large blocks of cHC on chromosomes 1, 9, 16, the acrocentric chromosomes and Y^7^, also referred to as ‘knobs’^5^.

The H3K9/HP1 pathway underlies the formation of cHC. A central event is the trimethylation of histone H3 at lysine 9 (H3K9me3) by histone methyltransferases (HMTs) Suv39h1 and Suv39h2 (refs 5, 8, 9). H3K9me3 serves as a docking place for the binding of heterochromatin protein 1 (HP1) isoforms, which results in chromatin compaction^5^. Subsequently, HP1 binds Suv4-20h1/2 HMTs, which trimethylate histone H4 at lysine 20 (H4K20me3) to further establish a compact chromatin structure^5^,^10^. Through an unidentified mechanism, H3K9me3 also facilitates the trimethylation of histone H3 at lysine 64 (H3K64me3), which has been suggested to stabilize cHC^11^,^12^. The H3K9/HP1 pathway is interwoven with the methylation of DNA, another mechanism for gene silencing prominent in cHC^5^,^10^. Together, all modifications eventually lead to the establishment of a condensed, transcriptionally repressed state that is epigenetically heritable through cell division.

In mammalian oocytes, the maternal genome is marked by high levels of histone lysine methylation, whereas in spermatozoa the paternal genome is compacted with small proteins named protamines^13^. Current knowledge of resolution of this epigenetic asymmetry in early mammalian embryos is mainly based on mouse models^1^. Paternal pHC in mouse spermatozoa and zygotes is largely devoid of canonical cHC marks^14^. Re-establishment of the canonical pHC configuration is not performed by the H3K9/HP1 pathway. Instead, during the earliest embryonic stages, maternally provided Polycomb repressive complex 1 (PRC1) localizes to paternal pHC, which subsequently becomes enriched for Polycomb repressive complex 2 (PRC2)-mediated trimethylation of histone H3 on lysine 27 (H3K27me3) (refs 3, 15). The core PRC1 complex contains an E3 ligase Ring1a/b, which interacts with one of the orthologues of the *Drosophila* posterior sex combs (Mel18, Bmi1 or Nspc1), a Polyhomeiotic orthologue (Phc1, Phc2 or Phc3) and a Polycomb orthologue (Cbx2, Cbx4, Cbx6, Cbx7 or Cbx8) (ref. 16). The PRC2 core complex contains one of the HMTs, Ezh1 or Ezh2, together with the regulatory subunits Suz12 and Eed^17^. In somatic cells, Polycomb complexes are known to regulate the formation of facultative heterochromatin, a type of heterochromatin that is able to undergo changes in configuration in the context of regulation of gene expression. Thus, in mouse preimplantation embryos, the paternal pericentric DNA temporarily assumes a facultative heterochromatin packaging, to circumvent the inactivity of the H3K9/HP1 pathway. The PRC1/2 pathway thereby operates as a transient backup mechanism for pHC formation^3^. During the eight-cell stage of mouse embryo development, the H3K9/HP1 pathway takes over again and the pHC of both parental origins gradually becomes equivalent for H3K9me3 (refs 3, 18). Other pHC-associated marks, such as H3K64me3 and H4K20me3, remain undetected in the paternal chromatin until after compaction and implantation, respectively^11^,^12^,^19^.

In this study, we addressed chromatin dynamics on cHC during human preimplantation embryo development. Our results identify striking differences with mouse: cHC in human embryos is not re-established by PRC1/2 action, but is transmitted and maintained by actors of the canonical H3K9/HP1 pathway. We show that human spermatozoa retain and transmit nucleosomes with cHC marks, such as H3K9me3, to the embryo. These paternal marks are subsequently bound by maternal HP1 and propagated over cell divisions. On the basis of this, we propose a model in which paternal cHC is transmitted intergenerationally.

## Results

### Pronuclear cHC localization in human zygotes

To determine the localization of cHC in human zygotes, pronuclear morphology was studied. Due to restrictions on the use of human embryos for research, we were limited to tripronuclear (3PN) zygotes and embryos resulting from 3PN zygotes to study embryos at Embryonic day (E) 1 and 2 (for overview of stages and source of embryos, see Supplementary Fig. 1a). Tripronuclear zygotes mostly originate from an oocyte that is fertilized by two spermatozoa, resulting in one maternal pronucleus and two paternal pronuclei^20^. Occasionally, when polar body extrusion fails, a 3PN zygote contains two maternal pronuclei and one paternal pronucleus. 3PN zygotes proceed through the first divisions normally and are capable of implantation, thus providing an ethically acceptable and relevant model for the first stages of preimplantation embryo development^21^,^22^. From E3 onwards, embryos developed from diploid (2PN) zygotes and donated for research were used (Supplementary Fig. 1a).

**Figure 1:**
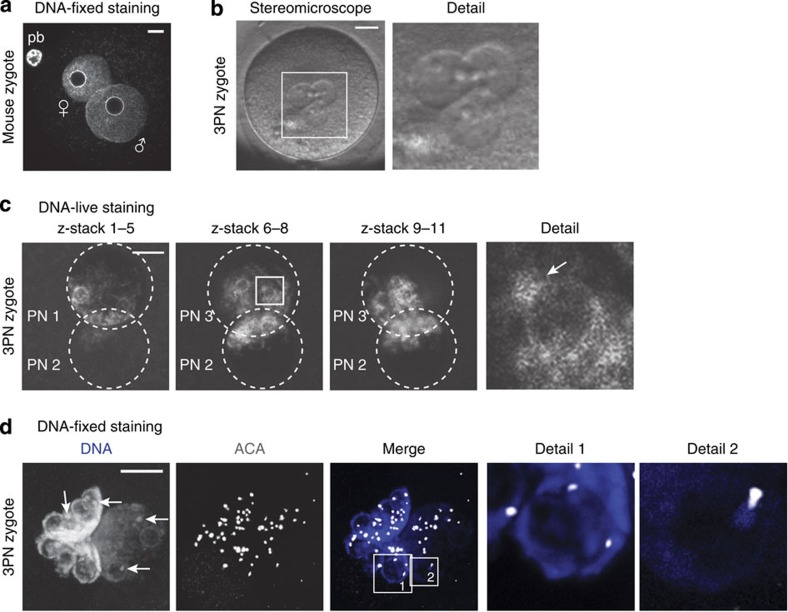
Pronuclear morphology of human zygotes differs from mouse zygotes. (**a**) Representative confocal image of a mouse zygote fixed 12–15 h post fertilization (G2 phase) with typical pronuclear morphology as observed by DNA staining (DAPI; *n*=10). Shown is a full projection of Z-sections. Pronuclei contain a ring-like structure called NPB and pHC can be observed as a ring with intense DAPI staining, surrounding the NPB. Paternal (♂) and maternal (♀) pronuclei are indicated, as is the polar body (pb). Scale bar, 10 μm. (**b**) Stereo micrograph of a human 3PN zygote showing typical pronuclear morphology. Detail shows a magnification of the boxed area. Scale bar, 20 μm. (**c**) Representative confocal image of a human zygote 18–20 h post insemination (G2 phase) with typical pronuclear morphology as observed by live staining with Hoechst 33342 (*n*=6). Shown are three projections of consecutive Z-sections through the three pronuclei. DNA inside each pronucleus is contracted into a crescent shape containing several ring-like structures and denser stained ‘knobs’. Detail shows a magnification of a single Z-section through the boxed ring-like structure with attached knob (arrow). Scale bar, 10 μm. (**d**) Representative confocal image of a human zygote fixed 18–20 h post insemination (G2 phase) with typical pronuclear morphology (*n*=10). Shown is a full projection of Z-sections with DNA staining (DAPI; blue) and immunolocalization of the centromeres (ACA; white). Pronuclear morphology is not affected by fixation and DNA is observed in the same crescent shape with ring-like structures and DAPI-intense knobs (arrows). Centromeres are localized preferentially on the ring or in close proximity to a knob. Details 1 and 2 show the magnifications of a single Z-section through the boxed ring-like structure and knob, respectively. Scale bar, 10 μm.

Pronuclear morphology of human zygotes differs from mouse zygotes. In late-stage mouse zygotes (G2 phase, also indicated as PN4/5 stage^23^), pronuclei are spherical and the DNA is spread throughout the pronucleus. A 4′-6-diamidino-2-phenylindole (DAPI)-intense ring-like structure around the nucleolar precursor body (NPB) contains the pHC^3^,^4^ (Fig. 1a). Human pronuclei also appear spherical under a stereomicroscope (Fig. 1b). However, when pronuclei are examined at G2 phase (18–20 h after fertilization^24^, Supplementary Fig. 1a) by DNA staining, the DNA content concentrates in the direction of the opposing pronucleus in both live and fixed conditions, resulting in a crescent shape (Fig. 1c,d). The NPBs are also contained in DAPI-rich ring-like structures, but are smaller and more numerous when compared with mouse NPBs at that stage. In addition, a few DAPI-rich knobs are observed, similar in appearance to cHC blocks in human somatic cells^5^. Using human autoantibodies against the centromeres (ACA), we show that the centromeres are situated in close proximity to both the rings and knobs (Fig. 1d). This confirms that these DAPI-rich chromatin domains are located pericentrically and are likely to contain cHC in human embryos.

### PRC1/2 are not associated with cHC in cleavage stages

**Figure 2:**
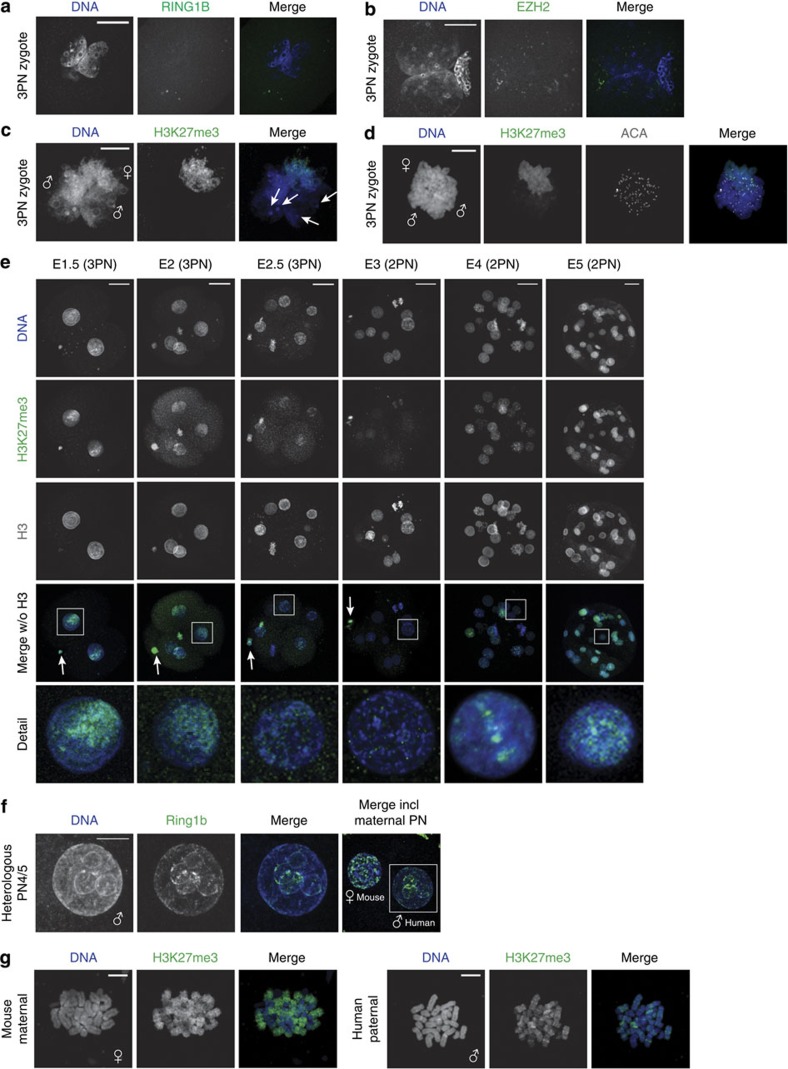
PRC1 and PRC2 are not associated with paternal cHC in cleavage stage human embryos. (**a**–**c**) Representative confocal images of human 3PN zygotes fixed at G2 phase. Shown are full projections of Z-sections. (**a**,**b**) Zygotes immunostained with PRC1 subunit RING1B (*n*=10) and PRC2 subunit EZH2 (*n*=5) antibodies (green). No signal is observed to localize to the pronuclei. Scale bars, 30 μm. (**c**) Immunolocalization of H3K27me3 (green) in a 3PN zygote (*n*=15). Paternal (♂) and maternal (♀) pronuclei are indicated. H3K27me3 is detected broadly on maternal chromatin and only very low levels are observed on paternal chromatin with no enrichment at DAPI-intense, heterochromatic rings or knobs (arrows). Scale bar, 10 μm. (**d**) Representative chromosome spread of a 3PN zygote (*n*=5) arrested at prometaphase showing immunolocalization of H3K27me3 (green) and centromeres (ACA; white). Paternal (♂) and maternal (♀) chromosomes are indicated. H3K27me3 is detected broadly on maternal chromosomes and no H3K27me3 enrichment is observed in the paternal pericentric regions. Scale bar, 10 μm. (**e**) Representative full projections of confocal Z-sections of human embryos at indicated stages (*n*=5–10 per embryonic stage). From embryonic day (E) 1 to 3, nuclei show an asymmetric staining pattern for H3K27me3 (green). Compared with the overall H3 levels as detected by a histone H3 antibody (white), H3K27me3 levels decrease gradually with each cell division. At E4, H3K27me3 is clearly detected throughout all nuclei. Arrows indicate the polar body, where H3K27me3 remains high. Detail shows a magnification of the boxed nucleus. Scale bars, 30 μm. (**f**) Mouse oocytes injected with human spermatozoa, fixed at PN4/5 stage (G2 phase). Shown is a representative confocal image of immunolocalization of Ring1b (green) in a heterologous zygote (*n*=30). Human paternal (♂) and mouse maternal (♀) pronuclei are indicated. The human paternal pronucleus assumes a mouse-like morphology and Ring1b is detected at distinct regions on the DAPI-intense ring around the nucleolar precursor bodies. Scale bar, 10 μm. (**g**) Representative chromosome spread from a heterologous zygote (*n*=5) arrested at prometaphase with immunolocalization of H3K27me3 (green). Human paternal (♂) and mouse maternal (♀) chromosomes are indicated. On human paternal chromosomes, H3K27me3 is detected and shows enrichment at distinct chromosome bands. Scale bars, 10 μm.

Protein localization of PRC1 and PRC2 subunits was performed by immunofluorescence in 3PN zygotes fixed at G2 phase. The enzymatic PRC1 subunit RING1B was not detected (Fig. 2a), whereas typical RING1B foci^25^ were observed in nuclei from hU2OS cells and human blastocysts (Supplementary Fig. 2a). Similar results were obtained for two other PRC1 subunits, RING1A and PHC2 (Supplementary Fig. 2b,c), as well as for the core components of PRC2, EZH2 (Fig. 2b) and EED (Supplementary Fig. 2d).

Subsequently, we characterized PRC2 activity by investigating the histone modification it catalyses—H3K27me3. Immunodetection of H3K27me3 in 3PN zygotes at the G2 phase revealed a clear asymmetry for H3K27me3 staining, with one pronucleus showing intense staining throughout, whereas in the other two pronuclei staining levels were barely detectable (Fig. 2c). These results are in accordance with our previous findings^20^ and those of Zhang *et al*.^26^ Since metaphase II chromosomes in oocytes show high levels of H3K27me3 (ref. 26) and in analogy to what has been described in mouse^15^,^27^ and other species^28^,^29^, we propose the pronucleus with strong H3K27me3 staining to be of maternal origin.

In mouse zygotes, paternal pHC becomes increasingly enriched for H3K27me3 by PRC2 activity during G2 phase^3^,^30^. In contrast, in human zygotes arrested at the prometaphase stage, H3K27me3 levels remained barely detectable on paternal chromosomes, also at pericentric regions (Fig. 2d). To determine whether PRC2 activity is upregulated during subsequent development, we performed immunostaining on whole-mount embryos of all stages of preimplantation development from the zygote at E1 to the blastocyst at E5 (for overview of stages and source of embryos see Supplementary Fig. 1a). On E1 and E2, H3K27me3 staining was restricted to one side of the nucleus, presumably marking the maternal chromatin. Overall levels of H3K27me3 decreased to become barely detectable at E3. From E4 onwards, H3K27me3 levels increased and the staining was distributed throughout the entire nucleus (Fig. 2e). To exclude differences caused by the triploid state of the embryos examined at E1 and E2, we compared embryos at E3 and E4 developed from normal, dipronuclear zygotes (Fig. 2e) with those developed from 3PN zygotes (Supplementary Fig. 2e) and observed no differences. The lack of PRC2 activity at E1–3 is thus unlikely to be related to the triploid state of the embryos investigated.

Next we analysed the abundance of mRNA transcripts for subunits of PRC1 and PRC2 by quantitative PCR with reverse transcription (RT–qPCR) in oocytes and embryos of eight developmental stages (for overview of stages, see Supplementary Fig. 1a). The mRNA expression of most PRC1 and PRC2 subunits followed similar patterns throughout preimplantation development: levels decreased from oocytes to the eight-cell stage at E3 and increased again from E3.5 onwards (Supplementary Fig. 3a), concomitant with the above-described increase in trimethylation levels of H3K27.

Although observed high levels of H3K27me3 on maternal chromatin indicates PRC2 activity at some point during the oocyte development, our failure to detect PRC1 and PRC2 at the paternal chromatin suggests the absence of these complexes in the zygote and the early embryo. Alternatively, our observations can also be explained by the inability of PRC complexes to target paternal chromatin. To differentiate between these possibilities, we injected human spermatozoa into mouse oocytes, resulting in heterologous zygotes (Supplementary Fig. 1b). When fixed at late G2 phase, the human paternal pronucleus was observed to have adopted a mouse-like pronuclear morphology. We detected Ring1b staining at the ring surrounding the NPBs (Fig. 2f). This shows that the mouse maternal Ring1b is able to access human paternal chromatin. When heterologous zygotes were arrested and fixed at prometaphase, H3K27me3 was detected in a banding pattern on human paternal chromatin in heterologous zygotes (Fig. 2g). This is in contrast to barely detectable levels on paternal prometaphase chromosomes in human zygotes (Fig. 2d). These results indicate that human sperm chromatin is intrinsically able to undergo PRC1/2 processing. Although we cannot completely rule out the possibility that the observed change in pronuclear structure positively affects the accessibility of paternal cHC in heterologous zygotes, the low abundance of PRC1 and PRC2 transcripts and the dilution of maternal H3K27me3 over divisions suggests an absence of active PRC1/2 in early human embryos. Altogether our results suggest that the PRC1/2 pathway does not have a role in paternal cHC establishment in human preimplantation embryos.

### H3K9me3 marks paternal chromatin on DAPI-rich regions

**Figure 3:**
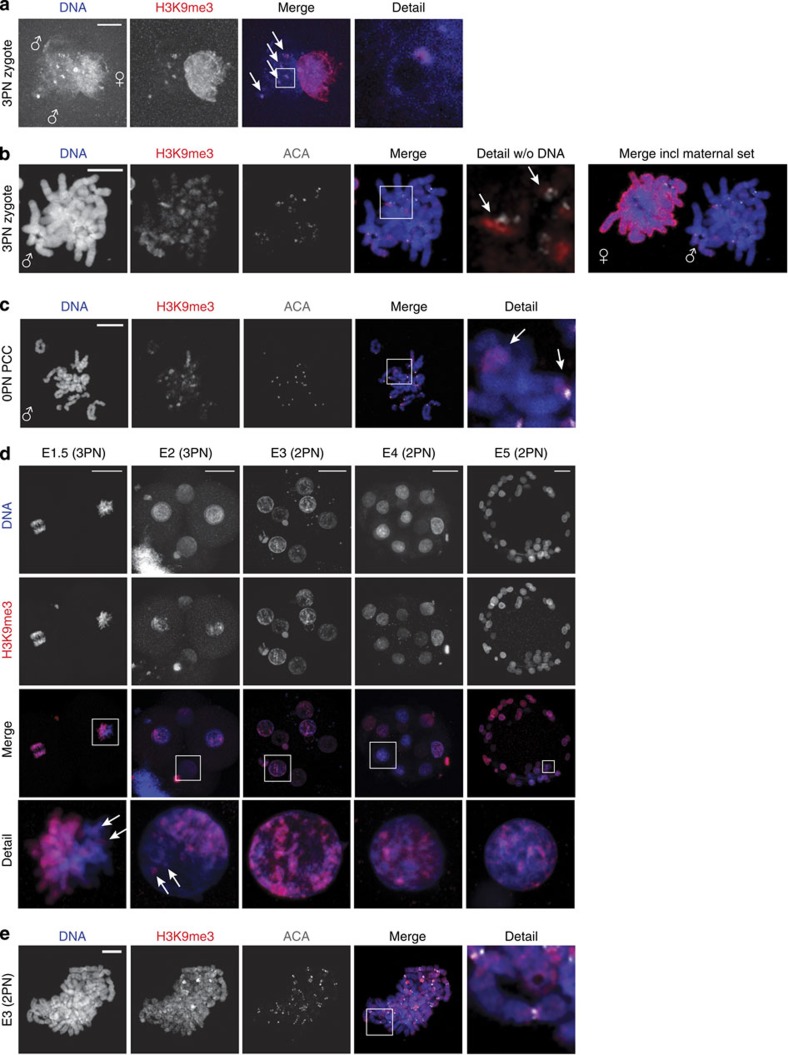
H3K9me3 is detected on DAPI-dense regions in paternal chromatin from cleavage stage human embryos. (**a**) Representative confocal image of a human 3PN zygote fixed at G2 phase (*n*=15). Shown is a full projection of Z-sections with immunolocalization of H3K9me3 (red). Paternal (♂) and maternal (♀) pronuclei are indicated. H3K9me3 is detected broadly on maternal chromatin and on paternal chromatin enrichment of H3K9me3 is detected at DAPI-intense, heterochromatic, regions (arrows). Detail shows a magnification of the boxed region. Scale bar, 10 μm. (**b**) Representative chromosome spread of a 3PN zygote arrested at prometaphase (*n*=30) showing the immunolocalization of H3K9me3 (red) and centromeres (ACA; white). Paternal (♂) and maternal (♀) chromosomes are indicated. H3K9me3 is detected broadly on maternal chromosomes. On paternal chromosomes, H3K9me3 is observed at inner centromeric regions (arrows) and some distinct chromosome bands (arrows). Detail shows a magnification of the boxed chromosomes. Scale bar, 10 μm. (**c**) Representative chromosome spread of a human oocyte 18–20 h post insemination with fertilization failure (0PN) and presence of paternal chromatids after PCC (*n*=10). Shown is the immunolocalization of H3K9me3 (red) and centromeres (ACA; white) on paternal chromatids. H3K9me3 is detected at distinct regions around the centromeres. Detail shows a magnification of the boxed chromatid (arrows indicate examples of the pericentromere and a H3K9me3 enriched band). Scale bar, 10 μm. (**d**) Representative full projections of confocal Z-sections of human embryos at indicated stages (*n*=5–10 per embryonic stage). On embryonic day (E) 1.5 and E2, H3K9me3 (red) is detected in an asymmetric pattern similar to the zygote. Levels on maternal chromatin remain high and arrows indicate H3K9me3 enriched regions on paternal chromatin. From E3 onwards, H3K9me3 was detected throughout the nucleus. Detail shows a magnification of the boxed nucleus. Scale bars, 30 μm. (**e**) Representative chromosome spread of an embryo at E3.5 arrested at the prometaphase of the fourth cleavage division with immunolocalization of H3K9me3 (red) and centromeres (ACA; white) (*n*=5). H3K9me3 was detected in a banding pattern on all chromosomes with enrichment at pericentric regions. Detail shows a magnification of the boxed chromosomes. Scale bar, 10 μm.

As the PRC1/2 pathway does not seem to be involved in the build-up of cHC in early human embryos, we investigated the canonical H3K9/HP1 pathway by immunostaining for H3K9me3. In human 3PN zygotes at the late G2 phase, maternal pronuclei are abundantly marked by H3K9me3, as described before^20^ (Fig. 3a). Although the signal in the paternal pronuclei was overall much lower, we did observe strong enrichment of H3K9me3 at the DAPI-rich knobs (Fig. 3a). This parental asymmetry in H3K9me3 was confirmed by the staining of prometaphase chromosome spreads: on paternal chromosomes, enrichment was detected between the centromeres and on a few chromosome bands (Fig. 3b).

Next we investigated if our observations for H3K9me3 on the paternal chromatin in post-S-phase human zygotes resulted from the *de novo* H3K9 methyltransferase activity during S-phase. In ~10% of *in vitro* fertilized (IVF) oocytes that fail to fertilize after IVF, a sperm cell has penetrated, but failed to activate the oocyte^31^. Such spermatozoa frequently undergo a condensation of the paternal chromatin into chromatids, called premature chromatid condensation (PCC). In oocytes with sperm-PCC, we detected strong H3K9me3 enrichment on paternal chromatids at pericentric regions and heterochromatic knobs (Fig. 3c), indicating that this mark is independent of DNA replication in S-phase.

To determine the fate of H3K9me3 on paternal chromatin in the zygote, we performed immunostaining of H3K9me3 on whole-mount embryos of all stages of preimplantation development (Supplementary Fig. 1a). In nuclei from E1 and E2 embryos, H3K9me3 staining was restricted to one side of the nucleus, presumably marking the maternal chromatin (Fig. 3d). In contrast to H3K27me3, levels on maternal chromatin remained high during these stages and DAPI-rich domains in the paternal chromatin remained positive for H3K9me3. From the eight-cell stage on, H3K9me3 was detected throughout the nucleus at DAPI-rich domains (Fig. 3d). These findings were confirmed by H3K9me3 staining of prometaphase chromosomes of eight-cell (E3) embryos arrested at the prometaphase stage of the fourth cleavage division; the difference between maternal and paternal chromosomes was no longer obvious (Fig. 3e). Taken together, these results show that H3K9me3 marks the paternal cHC in the zygote and suggest that this modification is maintained during subsequent cleavage divisions.

### H3K9me3 marks satellite DNA repeats in human spermatozoa

**Figure 4:**
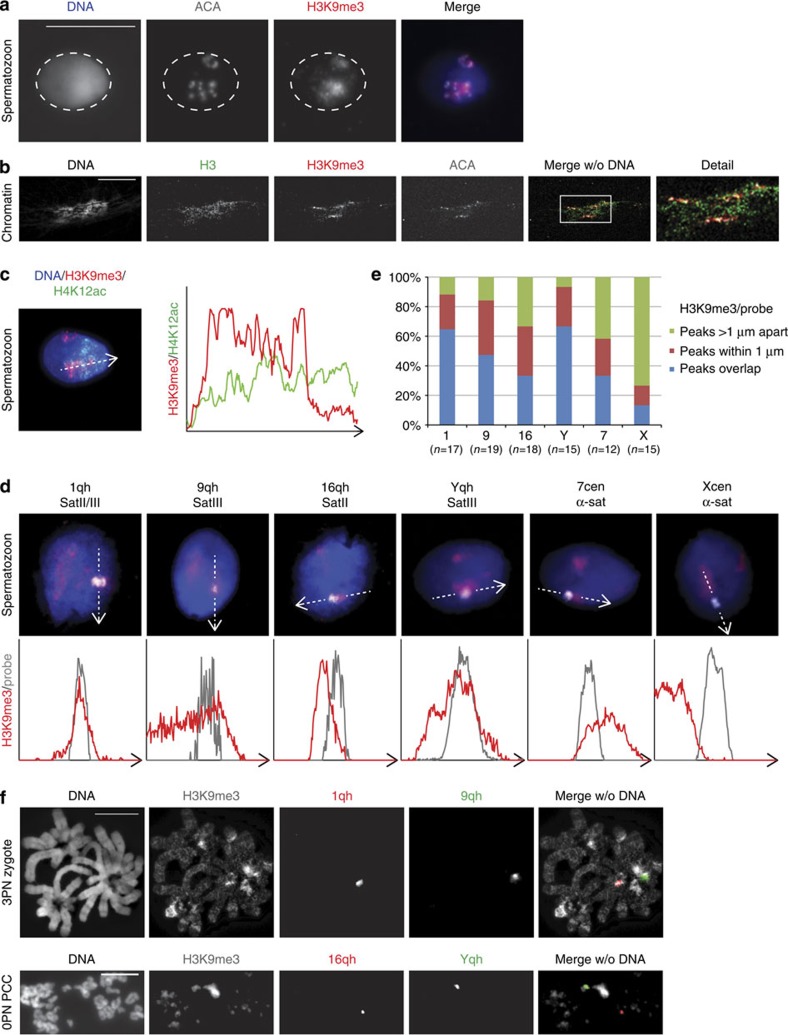
H3K9me3 marks satellite DNA sequences at cHC regions on paternal chromatin in spermatozoa and zygotes. (**a**) Representative confocal image of a human *in vitro* decondensed spermatozoon. Shown is a full projection of Z-sections with the immunolocalization of H3K9me3 (red) and centromeres (ACA; white) (*n*=100). Enrichment of H3K9me3 surrounding centromeres suggests pericentric localization. Dotted line indicates the nucleus of the spermatozoon. Scale bar, 10 μm. (**b**) Representative immunofluorescent image of human spermatozoa subjected to extreme *in vitro* decondensation. Chromatin contained in a nucleosomal structure was identified using a histone H3 antibody (green), in combination with immunolocalization of H3K9me3 (red) and centromeres (ACA; white) (*n*=100). H3K9me3 is detected directly neighbouring ACA signal, indicating pericentric localization. Scale bar, 20 μm. (**c**) Full projection of Z-sections of a human *in vitro* decondensed spermatozoon with immunolocalization of H3K9me3 (red) and H4K12ac (green) (full set of single-channel images in Supplementary Fig. 4). Graph shows the distribution of fluorescent intensities along the line (**c**) for H3K9me3 (red) and H4K12ac (green) in arbitrary units. (**d**) Codetection of H3K9me3 (red) and (peri)centric repeat sequences (white) in *in vitro* decondensed human spermatozoa (*n*=10–12) by immuno-FISH. DNA probes detecting satellite (Sat) DNA II/III repeat sequences at heterochromatic knobs on chromosomes 1, 9, 16 and Y and α satellite DNA sequences at centromeric locations (chromosomes 7 and X) are used. Shown are the representative merged images of a single Z-section through the probe signal (full set of single-channel images in Supplementary Fig. 5). Graph shows the distribution of fluorescent intensities along the line for H3K9me3 (red) and probe signal (grey) in arbitrary units. Scale bar, 5 μm. (**e**) Distribution of sperm cells according to their classification based on their profiles shown in **d**. (**f**) Codetection of H3K9me3 and (peri)centric repeat sequences by immuno-FISH on chromosome spreads. Upper panel: representative chromosome spread from the paternal chromosome set of a 3PN zygote arrested at prometaphase hybridized with DNA probes detecting satellite II/III sequences at chromosomes 1 (1qh, red) and 9 (9qh, green) (*n*=5). Lower panel: representative chromosome spread from paternal chromatids (PCC) in an unfertilized oocyte (0PN; *n*=7) hybridized with DNA probes detecting satellite II/III sequences at chromosomes 16 (16qh, red) and Y (yqh, green). DNA probes co-localize with areas of strong H3K9me3 (white) enrichment. Scale bars, 10 μm.

In mouse spermatogenesis, pHC becomes devoid of H3K9me3 as elongation is completed and the mark is also not detected on the paternal chromatin in the zygote directly after fertilization^14^,^27^. However, in human spermatozoa, H3K9me3 enrichment has been observed at the pericentric region of chromosome 16 using chromatin immunoprecipitation-PCR^32^. To further investigate the presence of H3K9me3 in mature human spermatozoa, we performed the immunolocalization of H3K9me3 and the centromeres (ACA) after *in vitro* decondensation. In virtually all spermatozoa, H3K9me3 was prominently detected around the centromeres (Fig. 4a). To investigate the location of retained H3K9me3 in more detail, human spermatozoa were subjected to extreme *in vitro* decondensation to obtain sperm chromatin in a fibre-like configuration. Whereas H3 was detected broadly, H3K9me3 was detected directly around the centromere, confirming its pericentric localization (Fig. 4b).

In mouse elongating spermatids, pHC domains that still contain residual H3K9me3-bearing nucleosomes also become enriched for nucleosomes with acetylation of histone H4 (refs 14, 33). Although these modifications appear to be mutually exclusive at the nucleosome level, pHC temporarily exists in a bivalent state that may contribute to the reprogramming process^33^. To investigate if cHC in mature human spermatozoa exists in a similar bivalent state, H4 acetylation was detected by immunofluorescence. Acetylation of lysines 5, 8 and 12 of histone H4 (H4K5ac, H4K8ac and H4K12ac) as well as a tetra-acetylated form of H4 (H4ac4) localized in a cap-like pattern, as described for human elongating spermatids^34^ (Supplementary Fig. 4). In contrast to mouse, the acetylated forms of H4 were not specifically enriched around the centromeres and H4K12ac was not confined to the H3K9me3-positive pHC domains (Fig. 4c).

To investigate if human DNA repeat sequences underlying cHC are marked by H3K9me3 in sperm cells, we performed a combination of immunofluorescent analysis of H3K9me3 and fluorescent *in situ* hybridization (immuno-FISH). We investigated α-satellite repeats located on chromosomes 7 and X and Sat II/III sequences of chromosomes 1, 9, 16 and Y. With the exception of the X α-satellite, all repeat sequences investigated were observed to localize partially or completely to the region enriched for H3K9me3 in the majority of sperm cells investigated (Fig. 4e,f, Supplementary Fig. 5). These results demonstrate that DNA repeat sequences that form the cHC in humans are enriched for H3K9me3 in sperm cells. To investigate if this is also the case after fertilization, we performed immuno-FISH on sperm-PCC in unfertilized oocytes and on paternal chromosomes of human zygotes arrested at prometaphase (Fig. 4g). Probe signals for Sat II and III sequences on chromosomes 1, 9, 16 and Y were observed to consistently co-localize with the H3K9me3-positive regions. The satellite III repeat region located to the knob on the long arm of the Y chromosome is completely marked by H3K9me3.

Thus, the DNA repeat sequences underlying cHC are enriched for H3K9me3 on paternal chromatin before and after fertilization. This suggests the delivery of these sequences in a canonical, heterochromatic conformation from the spermatozoon to the embryo.

### Sperm contributes modified histones marking cHC to zygotes

**Figure 5:**
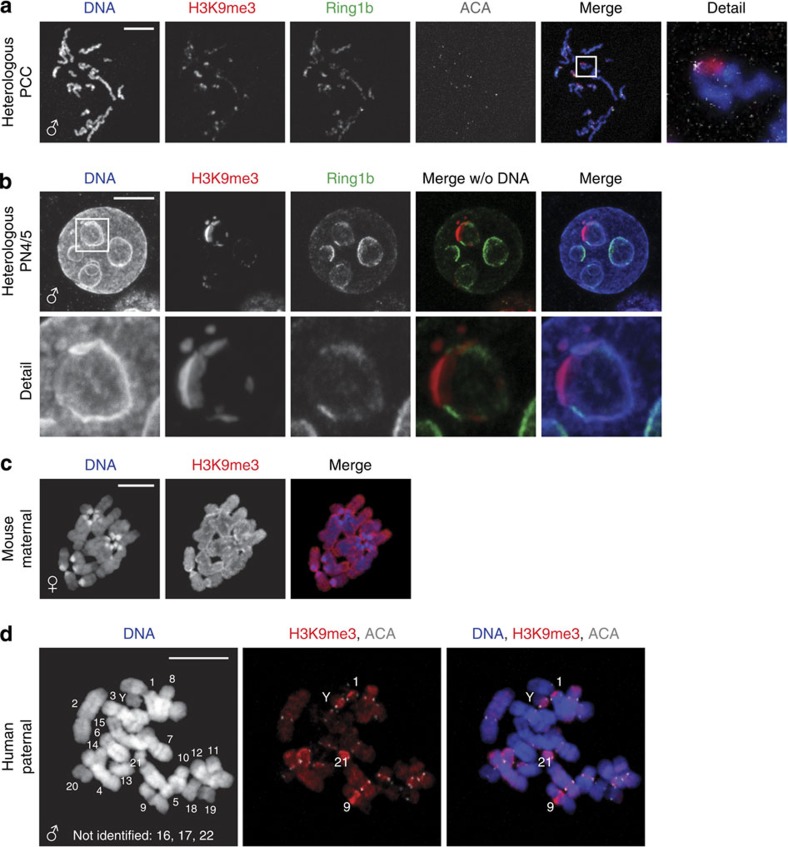
H3K9me3 on paternal embryonic chromatin originates from the human spermatozoon. To assess the origin of H3K9me3 on human paternal chromatin, human spermatozoa were injected into mouse oocytes. (**a**) Confocal analysis of fixed mouse oocytes 22 h after heterologous ICSI with heat-inactivated human sperm (*n*=30). Shown is a full projection of Z-sections containing paternal chromatids (♂) with immunolocalization of H3K9me3 (red) and Ring1b (green). H3K9me3 is clearly detected at pericentric regions on each chromatid. Scale bar, 10 μm. (**b**) Representative confocal image of the paternal pronucleus of a heterologous zygote fixed 12–15 h after injection (G2 phase; *n*=30). Shown is a full projection of Z-sections with immunolocalization of H3K9me3 (red) and Ring1b (green). The human paternal pronucleus (♂) assumes a mouse-like morphology (PN4). H3K9me3 and Ring1b are detected in a non-overlapping fashion at distinct regions at the DAPI-intense ring around the nucleolar precursor bodies. Detail shows a magnification of the boxed ring. Scale bar, 10 μm. (**c**,**d**) Representative chromosome spread of a zygote resulting from heterologous ICSI arrested at prometaphase (*n*=30). Paternal (♂) and maternal (♀) chromosomes are indicated. Scale bars, 10 μm. (**c**) Mouse maternal chromosomes with immunolocalization of H3K9me3 (red), showing H3K9me3 ubiquitously on maternal chromosomes. (**d**) On paternal chromosomes, the immunolocalization of H3K9me3 (red) and centromeres (ACA; white) revealed enrichment of H3K9me3 at the inner centromere of all chromosomes. Partial karyotyping of the paternal chromosomes identified strong enrichment on heterochromatin knobs on chromosomes 1, 9, 13, 14, 21 and Y. Chromosomes 16, 17 and 22 were not unambiguously identified in this spread.

Instead of the suggested inheritance of modified histones, our findings might also be explained by a scenario wherein paternally inherited nucleosomes are replaced by maternal ones, which are subsequently modified by maternal SUV39H1/2 activity. In mouse oocytes, the Suv39h-mediated modification of H3K9 is not active on paternal cHC^3^. Any H3K9me3 observed on human paternal chromatin after heterologous ICSI in mouse oocytes would thus be of sperm origin. First we injected heat-inactivated human spermatozoa (for experimental set-up of heterologous ICSI, see Supplementary Fig. 1b), which have lost the ability to activate the oocyte^35^ and directly undergo PCC after injection. In mouse oocytes with human sperm-PCC, we detected distinct H3K9me3-rich regions on the human paternal chromatids (Fig. 5a), identical to sperm-PCC in human oocytes (Fig. 3c). Next we injected normal human spermatozoa (Supplementary Fig. 1b) and investigated zygotes at the late G2 phase. The human paternal pronucleus exhibited distinct domains of H3K9me3 on the DAPI-rich rings around the NPBs (Fig. 5b), whereas this mark is absent from mouse paternal chromatin at this stage^3^. PRC1 is not targeted to the H3K9me3-positive domains, as these two types of heterochromatin do not overlap on both PCC and the paternal pronucleus at G2 (Fig. 5a,b). This reinforces the previous findings that H3K9me3 impairs PRC1 targeting^3^. In heterologous zygotes arrested at prometaphase, the H3K9me3 staining pattern was similar to the one observed on human 3PN zygotes. Distinct H3K9me3-positive bands were observed on human paternal chromosomes (Fig. 5c). Chromosome morphology of these heterologous spreads was generally good, and in some cases allowed (partial) karyotyping based on chromosome size and DAPI-banding pattern (Fig. 5d). Strong enrichment of H3K9me3 on heterochromatin knobs was specifically observed on chromosomes 1, 9, 13, 14, 16, 21 and Y, on regions known to contain satellite II and III DNA repeats.

Altogether these data suggest that the H3K9me3 in human spermatozoa is retained during the remodelling that occurs in a mouse oocyte after gamete fusion. On the basis of our previous observations^36^ and the data we presented here, we assume this also to be the case in human zygotes. Therefore, our results indicate a sperm origin of these modified histones in paternal embryonic chromatin.

### Maternal machinery reads and maintains paternal cHC

**Figure 6:**
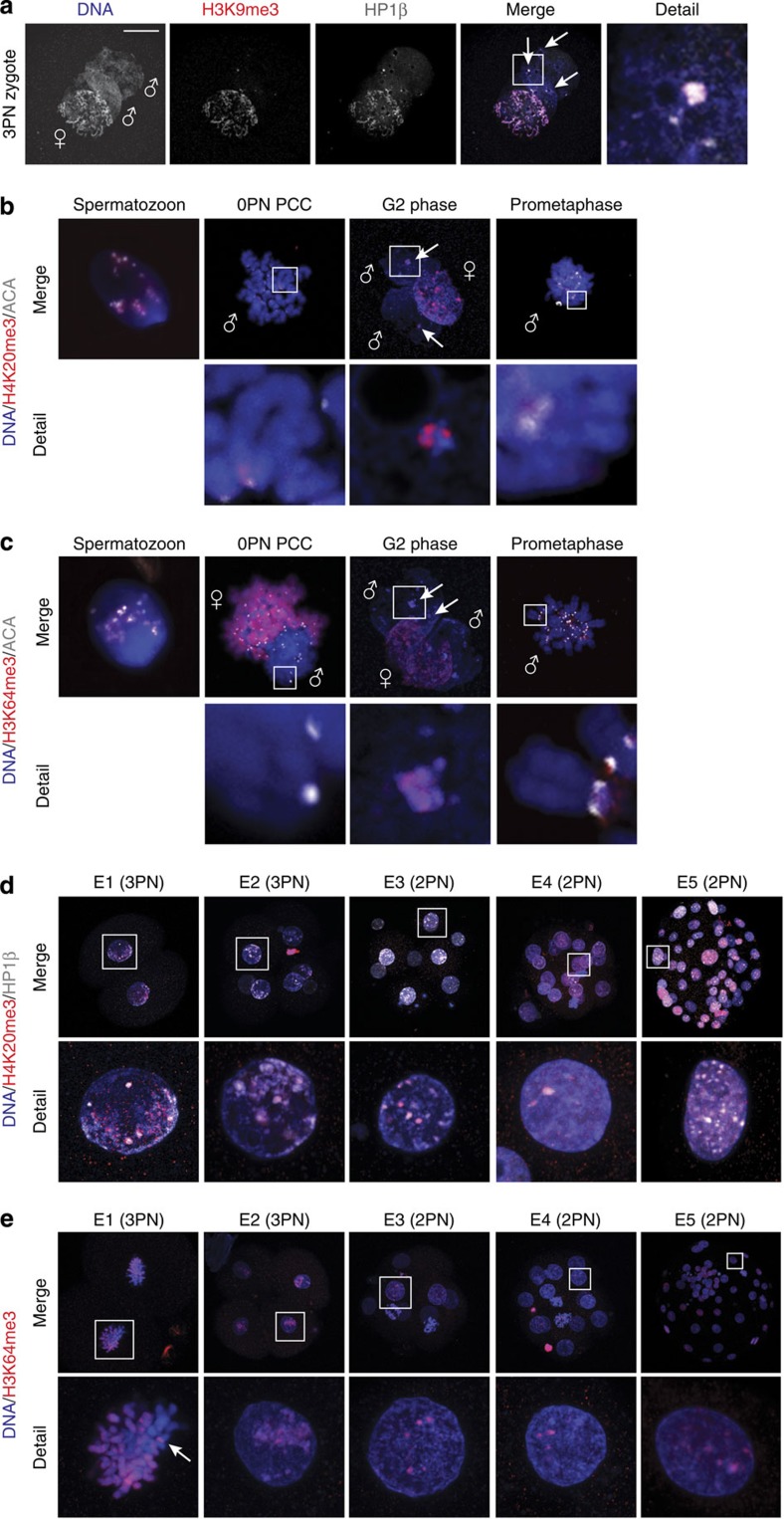
Paternal cHC marked by H3K9me3 also contains HP1β, H4K20me3 and H3K64me3. (**a**) Representative confocal image of a human 3PN zygote fixed 18–20 h post insemination with immunostaining of H3K9me3 (red) and HP1β (white) (*n*=5). Paternal (♂) and maternal (♀) pronuclei are indicated. Detail shows a magnification of the boxed region. HP1β was detected broadly on the maternal pronucleus and on heterochromatin knobs on the paternal pronucleus (arrows and detail), co-localizing with H3K9me3 staining. Scale bar, 10 μm. (**b**,**c**) Representative merged confocal images of a human *in vitro* decondensed spermatozoon, a human 0PN oocyte with PCC, a human 3PN zygote at G2 phase and a human 3PN zygote arrested at prometaphase (*n*=5–10 per embryonic stage), all immunostained for (**b**) H4K20me3 (red) and centromeres (ACA; white (excluding G2 phase)) or (**c**) H3K64me3 (red) and centromeres (ACA; white (excluding G2 phase)). Shown are full projections of Z-sections. Paternal (♂) and maternal (♀) chromatin is indicated. Details show the magnifications of boxed regions. Full set of single-channel images in Supplementary Fig. 5. Both H4K20me3 and H3K64me3 were detected at pericentric regions in all stages, comparable to H3K9me3 and HP1β, but H3K64me3 staining was less intense in spermatozoa and 0PN PCC. (**d**,**e**) Dynamics of H4K20me3, HP1β and H3K64me3 during human preimplantation embryo development. Representative merged confocal images of human embryos at indicated developmental stages (*n*=5–10 per embryonic stage) with immunolocalization of (**d**) H4K20me3 (red) and HP1β (white) or (**e**) H3K64me3 (red). Shown are the representative merged full projections of confocal Z-sections. Detail shows magnification of the boxed nucleus. Full set of single-channel images in Supplementary Fig. 6. On embryonic day (E) 1 and E2, all antibodies are detected in an asymmetric pattern similar to their expression the zygote. On condensed chromosomes, paternal H3K64me3 knobs are clearly visible (arrow). Whereas HP1β is observed strongly and throughout the nucleus from E3 onwards, H4K20me3 and H3K64me3 are more confined to DAPI-intense regions.

As described, the H3K9me3-binding HP1 proteins (isoforms α and β) and histone modifications H4K20me3 and H3K64me3 are additional markers of cHC. To investigate the extent of cHC formation on the paternal chromatin, we determined the dynamics of these additional markers by immunofluorescent analysis in spermatozoa, unfertilized oocytes with PCC, 3PN zygotes and all stages of preimplantation development. As described in mouse^3^, we also failed to detect HP1β in *in vitro* decondensed human spermatozoa (data not shown). However, in contrast to mouse, both HP1α and HP1β were readily detected at the DAPI-rich knobs in paternal pronuclei, co-localizing with H3K9me3 (Fig. 6a and Supplementary Fig. 6a). HP1β dynamics during embryo development followed the same pattern as H3K9me3, with asymmetric staining in the nucleus at E1 and E2 (Fig. 6d and Supplementary Fig. 7a). From E3 onwards, staining was observed at DAPI-rich regions throughout the nucleus.

H4K20me3 was detected around the centromeres in decondensed human spermatozoa, similar to H3K9me3. After fertilization, H4K20me3 was detected ubiquitously in the maternal chromatin, on DAPI-rich knobs at G2 phase in the paternal pronucleus and in the paternal pericentric regions in PCC and prometaphase chromosomes (Fig. 6b and Supplementary Fig. 6b). From the two-cell stage onwards, H4K20me3 remained detectable in a pattern similar to H3K9me3, but staining appeared more constricted to the DAPI-rich regions (Fig. 6d and Supplementary Fig. 7a).

H3K64me3 was observed at barely detectable levels in decondensed human spermatozoa. In unfertilized oocytes with paternal PCC, H3K64me3 was detected strongly in the maternal chromatin, but barely in the paternal chromatin. H3K64me3 levels appeared to increase after S-phase, as staining was observed on DAPI-rich knobs at G2 phase in the paternal pronucleus and prometaphase chromosomes (Fig. 6c and Supplementary Fig. 6b). From the two-cell stage onwards, H3K64me3 followed a pattern similar to H4K20me3 (Fig. 6e and Supplementary Fig. 7b).

These findings show that, in contrast to mouse, paternal cHC in human preimplantation stage embryos contains a full suite of canonical cHC markers, all of them grouped in the H3K9/HP1 pathway. Since we found that maternal HP1 binds sperm-derived H3K9me3 and that H3K64me3 is increased at H3K9me3-positive regions, our data also show that the modified histones contributed by the sperm cell enable further establishment and maintenance of cHC by the maternal machinery.

## Discussion

The current work identifies three major mechanistic differences in paternal cHC build-up between mouse and human: (1) human paternal cHC is transmitted from the spermatozoon to the zygote in a canonical conformation, which is subsequently propagated by the H3K9me3/HP1 pathway, (2) in humans, paternal embryonic cHC remains under control of the H3K9/HP1 pathway and cHC is characterized by hallmarks as H4K20me3 and H3K64me3 much earlier in development than in mouse and (3) a PRC1/2-mediated back-up mechanism, as described in mouse^3^, does not seem to play a role.

**Figure 7:**
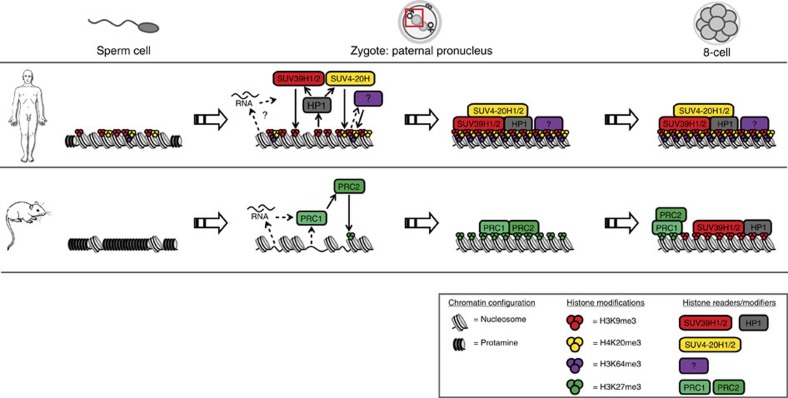
The build-up of paternal cHC in human embryos is directed by the H3K9/HP1 pathway and primed by sperm-derived histone modifications. Schematic representation of our results and conclusions and comparison with previous findings in mouse^3^. Whereas in mouse embryos, the re-establishment of paternal cHC is directed by the PRC1/2 pathway, in human embryos modified histones originating from the spermatozoon allow the build-up and maintenance of cHC by the canonical H3K9/HP1 pathway.

On the basis of our results, we propose an intergenerational model for cHC build-up in the early human embryo (Fig. 7): nucleosomes bearing H3K9me3 and H4K20me3 are transmitted by the spermatozoon and demarcate cHC domains in the zygote. H3K9me3 is detected and bound by maternally provided HP1 proteins, which in turn bind the SUV39H1/2 and SUV4-20H1/2 KMTs, enabling the propagation of these marks. Low levels of H3K64me3 present in the sperm cell can contribute to the feedback loop that reinforces further heterochromatinization^12^. Together this results in the epigenetic propagation of the chromatin conformation of paternal cHC to the next generation.

In mouse, only 1% of sperm DNA remains histone associated^37^. Although transferred to the embryo^14^, this low quantity of paternally inherited nucleosomes might not allow similar protein-based inheritance of heterochromatic DNA. Instead, paternal heterochromatic build-up is initiated through action of PRC1 (ref. 3). Recent findings indicate that the histone demethylase Kdm2b binds to unmethylated CpG islands and directly recruits a subset of PRC1 complexes to chromatin in pluripotent stem cells^38^. This or an analogous binding cascade could enable a DNA-based paternal heterochromatin build-up in mouse embryos^38^,^39^. Thus, whereas mouse embryos seem to depend on sperm DNA sequence and a maternal store of Polycomb proteins to re-establish paternal cHC, in human embryos cHC is inherited from the sperm cell in the canonical configuration.

In mouse, Polycombs are key to regulation of genes associated with development^40^. Therefore, our failure to detect PRC1/2 activity in human cleavage stage embryos is remarkable. However, immunofluorescence is not sensitive enough to detect possible presence and maintenance of H3K27me3 at a gene level. Genome-wide chromatin analysis of human sperm cells previously identified H3K27me3 enrichment at developmental regulators^32^,^41^. If transferred to the embryo in a similar fashion as H3K9me3, these marks might be enough to prevent inappropriate gene expression. A global decline in H3K27me3 levels during preimplantation development has also been described in bovine and porcine cleavage embryos, in which the embryonic stage with the lowest levels of H3K27me3 coincided with EGA^42^. Our findings are consistent with this, as the major wave of human EGA occurs around the eight-cell stage^43^. The global loss of H3K27me3 may thus be required for EGA in species where EGA occurs later than in mouse^42^.

In mouse embryos, the transcription of major satellites, especially from the paternal genome, has recently been reported to be crucial for rearrangement of pHC^4^. In this light, it is interesting that we observed strong H3K9me3 enrichment on satellite II/III DNA repeats on paternal chromatin in both spermatozoa and zygotes. Sat III repeat sequences are primate specific^44^ and long non-coding Sat III transcripts have been implicated in developmental regulation^45^. As long non-coding RNAs contribute to heterochromatin assembly during female X chromosome inactivation, Sat III transcripts may have a similar function in cHC maintenance^46^. It remains to be determined if such RNA-based mechanisms are needed for cHC organization in human embryos or if the self-sustaining loop of the full H3K9/HP1 pathway is sufficient.

During mammalian spermiogenesis, histones are replaced by protamines. In humans, 5–15% of the DNA, encompassing specific genes, as well as (peri)centromeric DNA, appear to be protected against this removal and retain a nucleosomal structure (for review, see ref. 47). Studies on human sperm have demonstrated that there is an increase in the nucleosome/protamine ratio when sperm from male factor subfertility patients are compared with sperm from fertile men, confirming incomplete chromatin remodelling during spermatid elongation^48^. Also, differences in the composition of retained nucleosomes exist between these groups, with a more dispersed pattern throughout the genome in sperm from subfertility patients^41^,^48^,^49^,^50^. As we show that sperm-inherited modified histones contribute to cHC formation in the human zygote, the variability observed in the retention of nucleosomes may interfere with cHC function in the zygote and impact on embryo developmental potential.

Frequently, the findings on chromatin dynamics in early mouse embryos are assumed to be universal for epigenetic reprogramming in mammalian embryos^51^,^52^. Our findings highlight the existence of divergent epigenetic developmental programs in mammals. Charting these differences will greatly improve our understanding of how heterochromatin features impact on embryo development.

## Methods

### Collection and culture of human gametes and embryos

All human surplus material was donated for research according to guidelines of the local ethical committee. Surplus embryos and oocytes were donated with patients’ written informed consent after approval by the Dutch Central Committee on Research Involving Human Subjects (CCMO—NL28739.000.09).

Ovarian stimulation, oocyte retrieval, IVF procedures and assessment of embryo morphology were performed as described^53^. Supernumerary good-quality embryos were cryopreserved. Cryopreservation was performed in a controlled rate freezer (in straws in culture medium with 1.5 M dimethyl sulfoxide. Straws were cooled to −6 °C before seeding and subsequently cooled to −40 °C at 0.3 °C min^−1^. Finally, the straws were cooled rapidly at −25 °C min^−1^ to −140 °C, before immersion in liquid nitrogen and storage in nitrogen vapour. After donation for research, thawing of embryos was performed at room temperature by consecutive washes in decreasing dimethyl sulfoxide concentrations in G-MOPS Plus medium (Vitrolife).

Oocytes that failed to fertilize after IVF (0PN) were obtained 18 h post insemination at embryonic day (E) 1. Tripronuclear (3PN) embryos were used to study embryo development from E1 to E2. Surplus cryopreserved preimplantation embryos of good quality were used to study embryonic developmental stages from E3 to E5 (for overview of stages, see Supplementary Fig. 1a). Embryo culture was performed in G1 Plus medium (Vitrolife) from E1 to E3 and thereafter in G2 Plus medium (Vitrolife) according to the manufacturer’s instructions. Human surplus spermatozoa were obtained from IVF patients meeting the criteria for normospermia (WHO, 2010). These sperm samples underwent routine workup by layering on a discontinuous silica gel gradient (PureSperm, Nidacon International) and centrifugation at 1,100 r.p.m. for 20 min. The resulting pellet was washed with G-IVF Plus medium (Vitrolife) at 1,600 r.p.m. for 10 min and kept in 0.5 ml G-IVF at 37 °C, 6% CO_2_ in air.

Human U2OS cells (a gift from Dr Kops^54^) were grown in Dulbecco’s Modified Eagle’s Medium (Sigma) supplemented with 10% fetal bovine serum (Sigma) and 100 U ml^−1^ penicillin, 100 μg ml^−1^ streptomycin (Invitrogen) and cultured at 37 °C in a humidified chamber in the presence of 5% CO_2_. Cells were plated on poly-D-Lysine-coated 12-mm coverslips, fixed with methanol at −20 °C for 10 min and then permeabilized with 0.2% Triton-X-100 in PBS.

### Antibodies

The following antibodies were used: rabbit polyclonal antibodies against H3K9me3 (1:500; Abcam ab8898), tetra-acetyl-Histone H4 (1:100; Upstate 06-598), H4K5ac (1:500; Abcam ab51997), H4K8ac (1:500; Upstate, 06-760), H4K12ac (1:500; Upstate 06-761), H4K20me3 (1:500 (ref. 55)), H3K64me3 (1:20 (ref. 11)) and H3K27me3 (1:200 (ref. 55)), mouse monoclonal antibodies against RING1A (1:200; Millipore 05-1362), RING1B (1:400 (ref. 56)), PHC2 (1:50 (ref. 57)), EZH2 (undiluted (ref. 58)), EED (undiluted (ref. 58)), HP1α (1:500; Euromedex 2HP-1H5-AS) and H3 (1:1,000; Active Motif 39763); rat monoclonal antibodies against H4K12ac (1:50; SciLight Biotechnology C2077001) and HP1β (1:500; Serotec MCA1946); and human anti-centromere antibodies (ACA; human centromere antiserum; 1:1,000, Fitzgerald Industries).

Primary antibodies were detected by labelling with the appropriate secondary antibodies conjugated with Alexa Fluor 488, 555, 594 or 633 (Invitrogen).

### Fixation and immunofluorescence of embryos

After removal of the zona pellucida by incubation with Acidic Tyrode’s Solution (Sigma), embryos were washed twice in G-MOPS, fixed for 15 min in 4% paraformaldehyde (PFA) in PBS (pH 7.4) and permeabilized with 0.2% Triton-X-100 in PBS for 15 min at room temperature. Embryos were incubated in blocking solution (0.1% Tween-20 in PBS containing 2% bovine serum albumin (BSA) and 5% normal goat serum) for 4 h at room temperature, followed by incubation with primary antibodies in blocking solution overnight at 4 °C. Embryos were washed three times for 20 min in 0.1% Tween-20 in PBS containing 2% BSA before application of secondary antibodies. These were diluted 1:200 in blocking solution and embryos were incubated for 1 h at room temperature, followed by three washing steps in 0.1% Tween-20 in PBS containing 2% BSA. Double antibody stainings were performed by mixing appropriate primary antibodies for simultaneous incubation, followed by detection with different secondary antibodies. Embryos were mounted on coverslips with Vectashield with 750 ng ml^−1^ DAPI for DNA counterstaining (Vector Laboratories)^3^.

For live staining of DNA in 3PN zygotes, zygotes were incubated in medium containing 1 μg ml^−1^ Hoechst 33342 and imaged directly.

To obtain chromosome spreads, embryos were incubated with 1.5 μg ml^−1^ colcemid (Invitrogen) for 8–16 h to arrest cells at prometaphase. After zona pellucida removal, arrested embryos were incubated in hyposolution (25% fetal calf serum in 0.5% sodium citrate) for 5 min and subsequently transferred to a drop of fixative (1% PFA with 0.2% Triton-X-100, pH 9.2) on a glass slide. After horizontal drying for 1 h, slides were washed with 0.08% Photo-Flo (Kodak) and air dried^59^. Slides with chromosome spreads were stored at −20 °C until use. Surface spread preparations were processed for immunofluorescence as described above without permeabilization.

For each embryonic stage and antibody investigated, five to ten embryos were analysed unless otherwise stated.

### Single oocyte and embryo RT–qPCR

mRNA levels were quantified in single oocytes and preimplantation embryos at the following eight developmental stages: metaphase II oocytes (E0; *n*=7); zygotes (E1; *n*=5); two-cell embryos (E1.5; *n*=5), four-cell (E2; *n*=4), eight-cell (E3; *n*=5), 12–16 cell (E3.5; *n*=5), morula (E4; *n*=4) and blastocyst (E5; *n*=5; Supplementary Fig. 1a). Human embryonic stem cells (WA01 (H1) Lot 11, WiCell Research Institute) were used as a control.

For RT–qPCR of single oocyte/embryos, the Taqman PreAmp Cells-to-Ct Kit (Applied Biosystems) was used according to the manufacturer’s protocol with minor adjustments^21^. The zona pellucida was removed from the oocytes and embryos by incubation in 0.1% protease (Sigma) in G-MOPS medium for 3 min, before washing in G-MOPS medium and PBS. Lysis was performed for 5 min in 20 μl of Taqman PreAmp Cells-to-Ct lysis solution and terminated by addition of 2 μl of stop solution. After 2 min of incubation the lysate was stored at −20 °C until further processing within 1 week. Small pieces of human embryonic stem cell colonies containing 500–1,000 cells were washed in PBS and transferred to 50 μl Taqman PreAmp Cells-to-Ct Lysis solution and terminated by addition of 5 μl stop solution. RNA was reverse transcribed to cDNA within an hour at 37 °C by adding 25 μl of 2 × RT Buffer and 2.5 μl of 20 × RT Enzyme Mix to each lysate, before inactivating the enzyme for 5 min at 95 °C. For sequence-specific preamplification of cDNA, Taqman Gene Expression Assays (Assays-on-demand, Applied Biosystems) were pooled and diluted 1:100 with 1 × TE buffer (10 mM Tris–HCl, 5 mM EDTA; pH 7.5) to a final concentration of 180 nM of each primer. The following assays (Applied Biosystems) were used: *ZP3* (Assay ID: Hs00610623_m1, amplicon size: 74 bp), *HPRT1* (Hs99999909_m1, 100 bp), *SOX2* (Hs01053049_s1, 91 bp), *OCT4* (Hs00999632_g1, 77 bp), *BMI1* (Hs00180411_m1, 105 bp), *MEL18* (Hs00810639_m1, 64 bp), *RING1A* (Hs00968517_m1, 71 bp), *RING1B* (Hs00200541_m1, 82 bp), *PHC2* (Hs00189460_m1, 114 bp), *CBX2* (Hs00364145_m1, 93 bp), *CBX7* (Hs00545603_m1, 54 bp), *CBX8* (Hs00221034_m1, 64 bp), *EZH1* (Hs00157470_m1, 65 bp), *EZH2* (Hs00544830_m1, 86 bp), and *EED* (Hs00537777_m1, 110 bp). Assays were selected to be exon spanning and to recognize most of the validated (Ref Seq) splice variants of each gene of interest.

To 12.5 μl of cDNA, 25 μl of Taqman PreAmp Master Mix and 12.5 μl of 0.2 × pooled Taqman Gene Expression Assays were added. After 10 cycles of preamplification (10 min at 95 °C, followed by 10 × 15 s at 95 °C and 4 min at 60 °C), the preamplified cDNA (50 μl) was diluted with 100 μl of 0.5 × TE buffer. qPCR was performed on an ABI Prism 7000 Sequence Detecting System (Applied Biosystems) using 10 μl of 2 × Taqman Gene Expression Master Mix, 1 μl of Taqman Gene Expression Assay and 5 μl of nuclease-free water added to 4 μl of diluted preamplified cDNA. The two-step cycling parameters were as follows: one cycle of 2 min at 50 °C, followed by one cycle of 10 min at 95 °C to activate the polymerase and 40 cycles of 15 s at 95 °C and 1 min at 60 °C (ref. 21). Results were analysed using Sequence Detection Software version 1.2.3 (Applied Biosystems) and expressed as cycle threshold values (Supplementary Fig. 3a). As a preamplification reaction of 10 cycles was performed, the detection limit of the qPCR was set at a cycle threshold value of 30 or less. Presence of a single PCR-product of expected amplicon size was verified by 2% agarose gel electrophoresis (Supplementary Fig. 3b).

### Heterologous intra cytoplasmic sperm injection

All institutional and national guidelines for the care and use of laboratory animals were followed and mouse experiments were approved by the local committee on animal experiments, DEC Consult. B6D2 F1 female mice (Harlan) were used as oocyte donors and superovulation was induced by intraperitoneal injection of 7.5 IU pregnant mare’s serum gonadotrophin (Intervet) followed by 7.5 IU human chorionic gonadotropin (Intervet) 48 h later^60^,^61^. Oocytes were isolated from the oviducts 13 h after human chorionic gonadotropin and cumulus cells were removed by brief incubation in G-MOPS medium containing 80 IU ml^−1^ hyaluronidase (Sigma). Thereafter, the oocytes were washed and kept until after injection in freshly prepared mem-alpha medium (Life Technologies), supplemented with 10% (v/v) fetal calf serum, 21 μM HEPES, 12.2 μM sodium lactate, 1 μM sodium pyruvate and 1 μM L-glutamin. Microinjection was performed using an inverted microscope equipped with an ICSI micromanipulation set-up (Narishige) and a piezo-actuated injector (Burleigh). A XYclone laser system (Hamilton Thorne) was used to breach the zona pellucida. Before installation, a small volume of mercury (Sigma) was inserted in an Straight Piezo Drill Micropipette (Humagen). Spermatozoa were used either directly after collection from the epididymis or, to prevent oocyte activation, after heat inactivation (incubation at 50 °C for 30 min (ref. 62)). For each injection series, an aliquot of spermatozoa was transferred to medium containing 12% polyvinyl pyrrolidone (Irvine Scientific). Spermatozoa were immobilized with a short piezo pulse applied to the neck piece. Injections were performed at room temperature. Injected oocytes were warmed to 37 °C and transferred after 5–10 min to G1 medium (Vitrolife) for culture at 37 °C, 5% CO_2_ in air. Oocytes injected with heat-inactivated spermatozoa (*n*=60) were fixed 22 h after injection with 4% PFA as described above. Oocytes injected with normal spermatozoa were either fixed with 4% PFA 12–15 h after injection (*n*=60) or transferred to medium containing 1.5 μg ml^−1^ colcemid and processed for chromosome spreads 7–9 h later as described above (*n*=60).

### *In vitro* sperm decondensation

Sperm head decondensation was achieved as described^48^, with some modifications. First, 5 μl of a spermatozoa suspension was brought on a glass slide and spread out using the side of the pipet tip. After drying, the slides were incubated in decondensation buffer (2.5 mM dithiothreitol (Sigma), 0.2% Triton-aX-100 (Sigma) in PBS) for 10 min, followed by incubation for 3, 4 and 5 min with 0.5% (v/v) heparin (5,000 U ml^−1^, LEO Pharma BV) in decondensation buffer. Subsequently, the slides were fixed in 4% PFA for 15 min, air dried, washed in Photo-Flo and air dried. Slides were processed for immunofluorescence immediately as described above, starting with two washes in PBS-T. For each sperm sample, the optimal decondensation time was determined by sperm head morphology and ACA antibody accessibility. Preparations with optimal decondensation were used for further analysis. The presence of each histone modification under investigation was assessed in at least 100 sperm cells with clearly distinguishable ACA staining, from at least three separate donors.

To obtain sperm DNA in a chromatin fibre-like structure, 5 μl of spermatozoa suspension was added to 80 μl decondensation buffer, as described above and incubated for 10–20 min. Heparin was added to a concentration of 0.5% (v/v) and incubated for 10 min. Of this decondensed spermatozoa suspension, 5 μl was brought on a glass slide, spread out using the side of the pipet tip and air dried. Slides were fixed in 4% PFA for 15 min and processed for immunofluorescence as described above.

### Immunofluorescence—FISH

For immuno-FISH on *in vitro* decondensed spermatozoa, slides were first processed for FISH. The DNA probes used were Satellite DNA II/III probes for chromosomes 1 (pUC1.77 (ref. 63)), 9 (pHuR98 (refs 64, 65)), 16 (pHuR195 (refs 64, 65)) and Y (RPN1305X (ref. 66)), and alpha satellite DNA probes for chromosomes 7 (pα7tl (ref. 67)) and X (pBamX5 (ref. 68)). Probes were fluorescently labelled using a BioPrime DNA labelling kit (Invitrogen), according to the manufacturer’s instructions. A hybridization mixture containing 1 ng μl^−1^ of labelled probe in 50% formamide, 10% dextran sulfate, 1% Tween-20 and 0.1 μg ml^−1^ Human Cot-1 DNA (Invitrogen) in 2 × standard saline citrate (SSC) was applied to each slide under a coverslip. Slides were denatured at 75 °C for 3 min and hybridization was performed in a humid box at 37 °C for 6 h. After hybridization, slides were washed in 2 × SSC/0.05% Tween-20 for 2 min at 42 °C, 0.4 × SSC for 6 min at 60 °C and in 2 × SSC/0.05% Tween-20 for 2 min at room temperature. The slides were rinsed once in PBS before proceeding to immunofluorescence of H3K9me3 as described above. Immuno-FISH was imaged simultaneously and for each probe co-localization for H3K9me3 and satellite DNA was measured in 10–12 randomly selected sperm cells from at least three separate donors.

For immuno-FISH on chromosome spreads of unfertilized oocytes or 3PN zygotes (five to seven per probe combination), slides were first processed for immunofluorescence of H3K9me3 as described above. Chromosome spreads were subsequently imaged and positions of chromosomes were recorded in the form of the XY-position of the microscope table. These coordinates were used to find the same chromosomes after FISH. Subsequently, FISH was performed as described^69^ for either Satellite DNA II/III probes for chromosome 1 (pUC1.77 (ref. 63)) in combination with 9 (pHuR98 (refs 64, 65)) or 16 (pHuR195 (refs 64, 65)) in combination with Y (RPN1305X (ref. 66)). Probes were hybridized overnight followed by post-hybridization washes and imaging of the FISH signals.

### Imaging and image analysis

Immunofluorescent images from whole-mount embryos, heterologous zygotes, chromosome spreads and immuno-FISH on *in vitro* decondensed spermatozoa were acquired using a Zeiss Axio Imager M2 confocal laser scanning microscope, equipped with four diode lasers (405, 488, 555, 639 nm), an Axiocam camera and Zen 2009 (Carl Zeiss) software. For embryos, we recorded Z-series of 5 μm slices and for chromosome spreads, we recorded Z-series of 0.5 μm slices. Images were processed with Image J (version 1.42n) and Adobe Photoshop CS3 software.

Imaging for immuno-FISH on chromosome spreads was performed on a Zeiss Axio Imager M1 microscope (Carl Zeiss), equipped with a CoolCube 1m camera (MetaSystems) and Isis FISH Imaging System software (version 5.4.7, MetaSystems). Images of immunofluorescence and FISH were obtained in two rounds of imaging and merged using Image J. With the ROI Manager in Image J, a selected area of one image was copied to the other images. Subsequently, selected areas were cropped and merged.

For visualization of co-localization between H3K9me3 and satellite DNA, the distribution of fluorescence intensities was plotted using the Straight lines selection tool in Image J. Subsequently, using the Analyze—Plot Profile tool, the distribution of fluorescence intensities along the selected line was obtained. Sperm cells were divided into three categories according to their profiles for H3K9me3 and satellite DNA: (1) profiles where the peak intensities for H3K9me3 and FISH signal completely overlap, (2) profiles where the peak intensities are within a 1 μm distance and 3) profiles where the peak intensities are further apart.

## Author contributions

E.B.B. and A.H.F.M.P. conceived the project and designed the experiments. C.W., G.W.v.d.H., C.E., M.T. and M.A. performed the experiments and C.W., G.W.v.d.H., M.A., A.H.F.M.P. and E.B.B. analysed the data and evaluated the results. W.M.B. provided mouse material and facilities for heterologous ICSI. J.S.E.L. was responsible for IVF patients and informed consent for donated human embryos. C.v.d.W., G.W.v.d.H. and E.B.B. wrote the manuscript. All authors contributed to manuscript revision and critical discussion.

## Additional information

**How to cite this article**: van de Werken, C. *et al*. Paternal heterochromatin formation in human embryos is H3K9/HP1 directed and primed by sperm-derived histone modifications. *Nat. Commun.* 5:5868 doi: 10.1038/ncomms6868 (2014).

## Supplementary Material

Supplementary Information Supplementary Figures 1-7

## References

[b1] Burton A. & Torres-Padilla M. E. Chromatin dynamics in the regulation of cell fate allocation during early embryogenesis. Nat. Rev. Mol. Cell Biol. 15, 723–735 (2014). 25303116 10.1038/nrm3885

[b2] Surani M. A. Reprogramming of genome function through epigenetic inheritance. Nature 414, 122–128 (2001). 11689958 10.1038/35102186

[b3] Puschendorf M. . PRC1 and Suv39h specify parental asymmetry at constitutive heterochromatin in early mouse embryos. Nat. Genet. 40, 411–420 (2008). 18311137 10.1038/ng.99

[b4] Probst A. V. . A strand-specific burst in transcription of pericentric satellites is required for chromocenter formation and early mouse development. Dev. Cell 19, 625–638 (2010). 20951352 10.1016/j.devcel.2010.09.002

[b5] Grewal S. I. & Jia S. Heterochromatin revisited. Nat. Rev. Genet. 8, 35–46 (2007). 17173056 10.1038/nrg2008

[b6] Jones K. W. & Corneo G. Location of satellite and homogeneous DNA sequences on human chromosomes. Nat. New Biol. 233, 268–271 (1971). 4107945 10.1038/newbio233268a0

[b7] Jones K. W. Satellite DNA. J. Med. Genet. 10, 273–281 (1973). 4129952 10.1136/jmg.10.3.273 PMC1013033

[b8] Peters A. H. . Loss of the Suv39h histone methyltransferases impairs mammalian heterochromatin and genome stability. Cell 107, 323–337 (2001). 11701123 10.1016/s0092-8674(01)00542-6

[b9] Peters A. H. & Schubeler D. Methylation of histones: playing memory with DNA. Curr. Opin. Cell Biol. 17, 230–238 (2005). 15780602 10.1016/j.ceb.2005.02.006

[b10] Muramatsu D., Singh P. B., Kimura H., Tachibana M. & Shinkai Y. Pericentric heterochromatin generated by HP1 protein interaction-defective histone methyltransferase Suv39h1. J. Biol. Chem. 288, 25285–25296 (2013). 23836914 10.1074/jbc.M113.470724 PMC3757193

[b11] Daujat S. . H3K64 trimethylation marks heterochromatin and is dynamically remodeled during developmental reprogramming. Nat. Struct. Mol. Biol. 16, 777–781 (2009). 19561610 10.1038/nsmb.1629

[b12] Lange U. C. . Dissecting the role of H3K64me3 in mouse pericentromeric heterochromatin. Nat. Commun. 4, 2233 (2013). 23903902 10.1038/ncomms3233

[b13] Balhorn R. A model for the structure of chromatin in mammalian sperm. J. Cell Biol. 93, 298–305 (1982). 7096440 10.1083/jcb.93.2.298 PMC2112839

[b14] van der Heijden G. W. . Transmission of modified nucleosomes from the mouse male germline to the zygote and subsequent remodeling of paternal chromatin. Dev. Biol. 298, 458–469 (2006). 16887113 10.1016/j.ydbio.2006.06.051

[b15] Santos F., Peters A. H., Otte A. P., Reik W. & Dean W. Dynamic chromatin modifications characterise the first cell cycle in mouse embryos. Dev. Biol. 280, 225–236 (2005). 15766761 10.1016/j.ydbio.2005.01.025

[b16] Simon J. A. & Kingston R. E. Mechanisms of polycomb gene silencing: knowns and unknowns. Nat. Rev. Mol. Cell Biol. 10, 697–708 (2009). 19738629 10.1038/nrm2763

[b17] Margueron R. & Reinberg D. The Polycomb complex PRC2 and its mark in life. Nature 469, 343–349 (2011). 21248841 10.1038/nature09784 PMC3760771

[b18] Merico V. . Epigenomic differentiation in mouse preimplantation nuclei of biparental, parthenote and cloned embryos. Chromosome Res. 15, 341–360 (2007). 17447149 10.1007/s10577-007-1130-5

[b19] Wongtawan T., Taylor J. E., Lawson K. A., Wilmut I. & Pennings S. Histone H4K20me3 and HP1alpha are late heterochromatin markers in development, but present in undifferentiated embryonic stem cells. J. Cell Sci. 124, 1878–1890 (2011). 21576353 10.1242/jcs.080721

[b20] van der Heijden G. W. . Parental origin of chromatin in human monopronuclear zygotes revealed by asymmetric histone methylation patterns, differs between IVF and ICSI. Mol. Reprod. Dev. 76, 101–108 (2009). 18481364 10.1002/mrd.20933

[b21] Avo Santos M. . A role for Aurora C in the chromosomal passenger complex during human preimplantation embryo development. Hum. Reprod. 26, 1868–1881 (2011). 21493633 10.1093/humrep/der111

[b22] Mantikou E. . Temporal and developmental-stage variation in the occurrence of mitotic errors in tripronuclear human preimplantation embryos. Biol. Reprod. 89, 42 (2013). 23863408 10.1095/biolreprod.113.107946

[b23] Adenot P. G., Mercier Y., Renard J. P. & Thompson E. M. Differential H4 acetylation of paternal and maternal chromatin precedes DNA replication and differential transcriptional activity in pronuclei of 1-cell mouse embryos. Development 124, 4615–4625 (1997). 9409678 10.1242/dev.124.22.4615

[b24] Feenan K. & Herbert M. Can ‘abnormally’ fertilized zygotes give rise to viable embryos? Hum. Fertil. (Camb) 9, 157–169 (2006). 17008268 10.1080/14647270600636269

[b25] Hernandez-Munoz I., Taghavi P., Kuijl C., Neefjes J. & van Lohuizen M. Association of BMI1 with polycomb bodies is dynamic and requires PRC2/EZH2 and the maintenance DNA methyltransferase DNMT1. Mol. Cell. Biol. 25, 11047–11058 (2005). 16314526 10.1128/MCB.25.24.11047-11058.2005 PMC1316945

[b26] Zhang A. . Dynamic changes of histone H3 trimethylated at positions K4 and K27 in human oocytes and preimplantation embryos. Fertil. Steril. 98, 1009–1016 (2012). 22818287 10.1016/j.fertnstert.2012.06.034

[b27] van der Heijden G. W. . Asymmetry in Histone H3 variants and lysine methylation between paternal and maternal chromatin of the early mouse zygote. Mech. Dev. 122, 1008–1022 (2005). 15922569 10.1016/j.mod.2005.04.009

[b28] Ross P. J. . Polycomb gene expression and histone H3 lysine 27 trimethylation changes during bovine preimplantation development. Reproduction 136, 777–785 (2008). 18784248 10.1530/REP-08-0045

[b29] Park K. E., Magnani L. & Cabot R. A. Differential remodeling of mono- and trimethylated H3K27 during porcine embryo development. Mol. Reprod. Dev. 76, 1033–1042 (2009). 19536841 10.1002/mrd.21061

[b30] Santenard A. . Heterochromatin formation in the mouse embryo requires critical residues of the histone variant H3.3. Nat. Cell Biol. 12, 853–862 (2010). 20676102 10.1038/ncb2089 PMC3701880

[b31] Zenzes M. T., de Geyter C., Bordt J., Schneider H. P. & Nieschlag E. Abnormalities of sperm chromosome condensation in the cytoplasm of immature human oocytes. Hum. Reprod. 5, 842–846 (1990). 2266159 10.1093/oxfordjournals.humrep.a137195

[b32] Hammoud S. S. . Distinctive chromatin in human sperm packages genes for embryo development. Nature 460, 473–478 (2009). 19525931 10.1038/nature08162 PMC2858064

[b33] Govin J. . Pericentric heterochromatin reprogramming by new histone variants during mouse spermiogenesis. J. Cell Biol. 176, 283–294 (2007). 17261847 10.1083/jcb.200604141 PMC2063955

[b34] De Vries M., Ramos L., Housein Z. & De Boer P. Chromatin remodelling initiation during human spermiogenesis. Biol. Open 1, 446–457 (2012). 23213436 10.1242/bio.2012844 PMC3507207

[b35] Chao S. B. . Heated spermatozoa: effects on embryonic development and epigenetics. Hum. Reprod. 27, 1016–1024 (2012). 22313867 10.1093/humrep/des005

[b36] van der Heijden G. W. . Sperm-derived histones contribute to zygotic chromatin in humans. BMC Dev. Biol. 8, 34 (2008). 18377649 10.1186/1471-213X-8-34 PMC2358879

[b37] Balhorn R., Gledhill B. L. & Wyrobek A. J. Mouse sperm chromatin proteins: quantitative isolation and partial characterization. Biochemistry 16, 4074–4080 (1977). 911755 10.1021/bi00637a021

[b38] He J. . Kdm2b maintains murine embryonic stem cell status by recruiting PRC1 complex to CpG islands of developmental genes. Nat. Cell Biol. 15, 373–384 (2013). 23502314 10.1038/ncb2702 PMC4078788

[b39] Casanova M. . Heterochromatin reorganization during early mouse development requires a single-stranded noncoding transcript. Cell Rep. 4, 1156–1167 (2013). 24055057 10.1016/j.celrep.2013.08.015

[b40] Posfai E. . Polycomb function during oogenesis is required for mouse embryonic development. Genes Dev. 26, 920–932 (2012). 22499591 10.1101/gad.188094.112 PMC3347790

[b41] Brykczynska U. . Repressive and active histone methylation mark distinct promoters in human and mouse spermatozoa. Nat. Struct. Mol. Biol. 17, 679–687 (2010). 20473313 10.1038/nsmb.1821

[b42] Bogliotti Y. S. & Ross P. J. Mechanisms of histone H3 lysine 27 trimethylation remodeling during early mammalian development. Epigenetics 7, 976–981 (2012). 22895114 10.4161/epi.21615 PMC3515017

[b43] Vassena R. . Waves of early transcriptional activation and pluripotency program initiation during human preimplantation development. Development 138, 3699–3709 (2011). 21775417 10.1242/dev.064741 PMC4074286

[b44] Jarmuz M., Glotzbach C. D., Bailey K. A., Bandyopadhyay R. & Shaffer L. G. The Evolution of satellite III DNA subfamilies among primates. Am. J. Hum. Genet. 80, 495–501 (2007). 17273970 10.1086/512132 PMC1821104

[b45] Sandqvist A. . Heterotrimerization of heat-shock factors 1 and 2 provides a transcriptional switch in response to distinct stimuli. Mol. Biol. Cell 20, 1340–1347 (2009). 19129477 10.1091/mbc.E08-08-0864 PMC2649261

[b46] Biamonti G. & Vourc’h C. Nuclear stress bodies. Cold Spring Harb. Perspect. Biol. 2, a000695 (2010). 20516127 10.1101/cshperspect.a000695 PMC2869524

[b47] Miller D., Brinkworth M. & Iles D. Paternal DNA packaging in spermatozoa: more than the sum of its parts? DNA, histones, protamines and epigenetics. Reproduction 139, 287–301 (2010). 19759174 10.1530/REP-09-0281

[b48] Ramos L. . Incomplete nuclear transformation of human spermatozoa in oligo-astheno-teratospermia: characterization by indirect immunofluorescence of chromatin and thiol status. Hum. Reprod. 23, 259–270 (2008). 18056059 10.1093/humrep/dem365

[b49] Zhang X., San Gabriel M. & Zini A. Sperm nuclear histone to protamine ratio in fertile and infertile men: evidence of heterogeneous subpopulations of spermatozoa in the ejaculate. J. Androl. 27, 414–420 (2006). 16474017 10.2164/jandrol.05171

[b50] Hammoud S. S. . Genome-wide analysis identifies changes in histone retention and epigenetic modifications at developmental and imprinted gene loci in the sperm of infertile men. Hum. Reprod. 26, 2558–2569 (2011). 21685136 10.1093/humrep/der192 PMC3157626

[b51] Probst A. V. & Almouzni G. Heterochromatin establishment in the context of genome-wide epigenetic reprogramming. Trends Genet. 27, 177–185 (2011). 21497937 10.1016/j.tig.2011.02.002

[b52] Fadloun A., Eid A. & Torres-Padilla M. E. Mechanisms and dynamics of heterochromatin formation during mammalian development: closed paths and open questions. Curr. Top. Dev. Biol. 104, 1–45 (2013). 23587237 10.1016/B978-0-12-416027-9.00001-2

[b53] Hohmann F. P., Macklon N. S. & Fauser B. C. A randomized comparison of two ovarian stimulation protocols with gonadotropin-releasing hormone (GnRH) antagonist cotreatment for in vitro fertilization commencing recombinant follicle-stimulating hormone on cycle day 2 or 5 with the standard long GnRH agonist protocol. J. Clin. Endocrinol. Metab. 88, 166–173 (2003). 12519847 10.1210/jc.2002-020788

[b54] Kwiatkowski N. . Small-molecule kinase inhibitors provide insight into Mps1 cell cycle function. Nat. Chem. Biol. 6, 359–368 (2010). 20383151 10.1038/nchembio.345 PMC2857554

[b55] Peters A. H. . Partitioning and plasticity of repressive histone methylation states in mammalian chromatin. Mol. Cell 12, 1577–1589 (2003). 14690609 10.1016/s1097-2765(03)00477-5

[b56] Atsuta T. . Production of monoclonal antibodies against mammalian Ring1B proteins. Hybridoma 20, 43–46 (2001). 11289226 10.1089/027245701300060427

[b57] Isono K. . Mammalian polyhomeotic homologues Phc2 and Phc1 act in synergy to mediate polycomb repression of Hox genes. Mol. Cell. Biol. 25, 6694–6706 (2005). 16024804 10.1128/MCB.25.15.6694-6706.2005 PMC1190356

[b58] Hamer K. M. . A panel of monoclonal antibodies against human polycomb group proteins. Hybrid. Hybridomics 21, 245–252 (2002). 12193277 10.1089/153685902760213859

[b59] van de Werken C. . A universal method for sequential immunofluorescent analysis of chromatin and chromatin-associated proteins on chromosome spreads. Chromosome Res. 21, 475–489 (2013). 23896649 10.1007/s10577-013-9373-9

[b60] Kimura Y. & Yanagimachi R. Intracytoplasmic sperm injection in the mouse. Biol. Reprod. 52, 709–720 (1995). 7779992 10.1095/biolreprod52.4.709

[b61] Baart E. B. . Reduced oocyte activation and first cleavage rate after ICSI with spermatozoa from a sterile mouse chromosome mutant. Hum. Reprod. 19, 1140–1147 (2004). 15044406 10.1093/humrep/deh184

[b62] Perry A. C., Wakayama T. & Yanagimachi R. A novel trans-complementation assay suggests full mammalian oocyte activation is coordinately initiated by multiple, submembrane sperm components. Biol. Reprod. 60, 747–755 (1999). 10026126 10.1095/biolreprod60.3.747

[b63] Cooke H. J. & Hindley J. Cloning of human satellite III DNA: different components are on different chromosomes. Nucleic Acids Res. 6, 3177–3197 (1979). 573470 10.1093/nar/6.10.3177 PMC327928

[b64] Tagarro I., Fernandez-Peralta A. M. & Gonzalez-Aguilera J. J. Chromosomal localization of human satellites 2 and 3 by a FISH method using oligonucleotides as probes. Hum. Genet. 93, 383–388 (1994). 8168808 10.1007/BF00201662

[b65] Moyzis R. K. . Human chromosome-specific repetitive DNA sequences: novel markers for genetic analysis. Chromosoma 95, 375–386 (1987). 3677921 10.1007/BF00333988

[b66] Lau Y. F. Detection of Y-specific repeat sequences in normal and variant human chromosomes using *in situ* hybridization with biotinylated probes. Cytogenet. Cell Genet. 39, 184–187 (1985). 4042685 10.1159/000132132

[b67] Waye J. S., England S. B. & Willard H. F. Genomic organization of alpha satellite DNA on human chromosome 7: evidence for two distinct alphoid domains on a single chromosome. Mol. Cell. Biol. 7, 349–356 (1987). 3561394 10.1128/mcb.7.1.349-356.1987 PMC365075

[b68] Willard H. F., Smith K. D. & Sutherland J. Isolation and characterization of a major tandem repeat family from the human X chromosome. Nucleic Acids Res. 11, 2017–2033 (1983). 6300789 10.1093/nar/11.7.2017 PMC325859

[b69] Baart E. B., Martini E. & Van Opstal D. Screening for aneuploidies of ten different chromosomes in two rounds of FISH: a short and reliable protocol. Prenat. Diagn. 24, 955–961 (2004). 15614916 10.1002/pd.1052

