# Supplementary material for: Paternal heterochromatin formation in human embryos is H3K9/HP1 directed and primed by sperm-derived histone modifications
Source: Nat Commun. 2014 Dec 18;5:5868. doi: 10.1038/ncomms6868 (PMC4284653; doi:10.1038/ncomms6868)
Supplement: Supplementary Information — Supplementary Figures 1-7 [file ncomms6868-s1.pdf]

Supplementary Figure 1

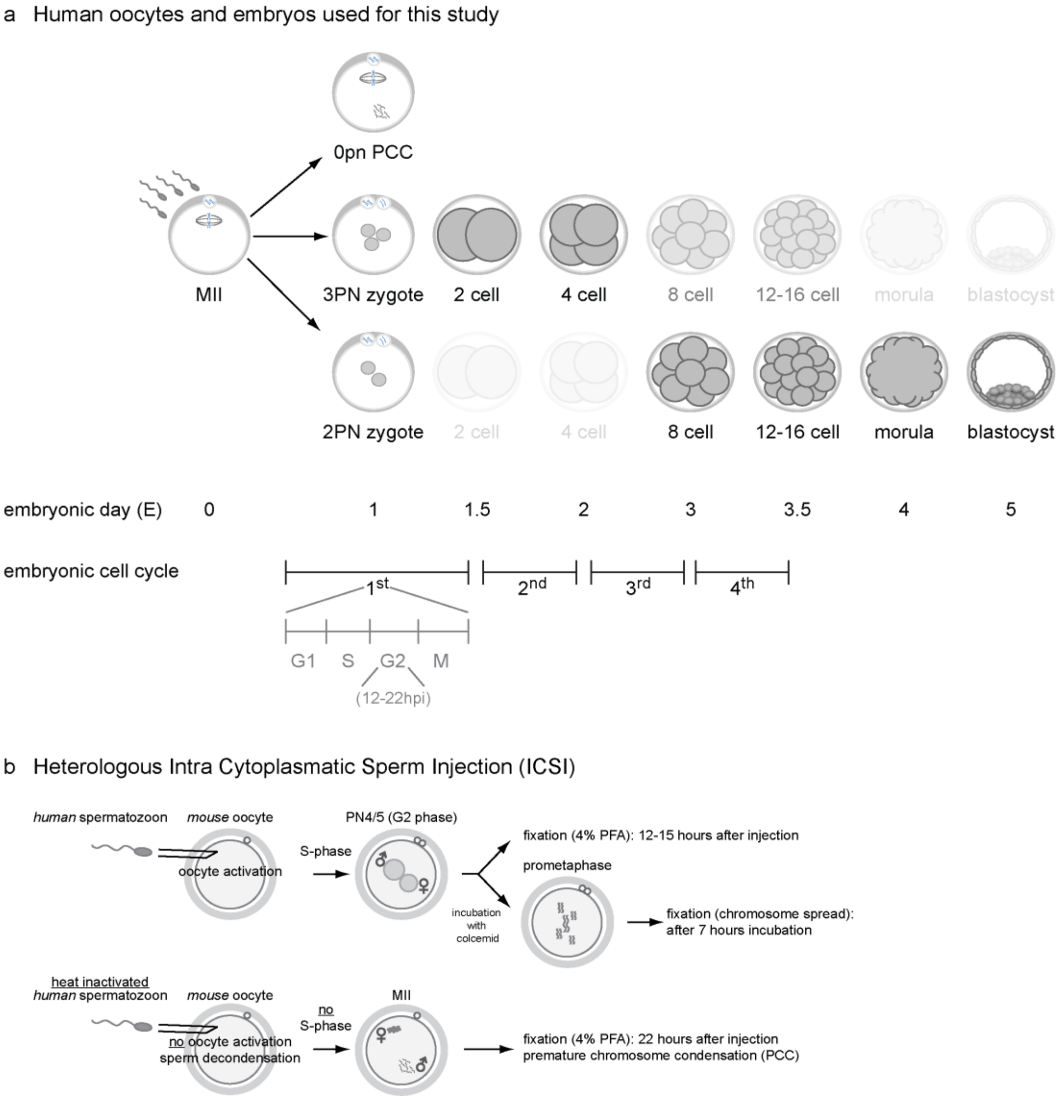

Developmental time-frame for human oocytes and pre-implantation embryos used in this study. (**a**) Schematic representation of the stages of oocyte and embryo development used in this study. Oocytes that failed to fertilize were processed on E1 and checked for the presence of paternal chromatids (PCC). Zygotes that displayed three pronuclei (3PN) were either processed directly, incubated in colcemid for prometaphase arrest or allowed to develop until E1 or E2 and processed subsequently. Good quality diploid (2PN) embryos that had been cryopreserved on E3 or E4 were thawed and processed immediately or allowed to develop to E4 or E5 and processed subsequently. (**b**) Schematic representation of heterologous ICSI experiments: mouse oocytes are injected with normal or heat-inactivated human spermatozoa. Oocytes injected with normal spermatozoa are either fixed 12-15 h post injection at the PN4/5 stage (corresponding to G2 phase) or prepared for chromosome spreads after incubation with colcemid to induce prometaphase arrest. Oocytes injected with heat inactivated spermatozoa fail to activate and remain at the metaphase II (MII) stage, while paternal chromatin is condensed into chromatids (PCC).

Supplementary Figure 2

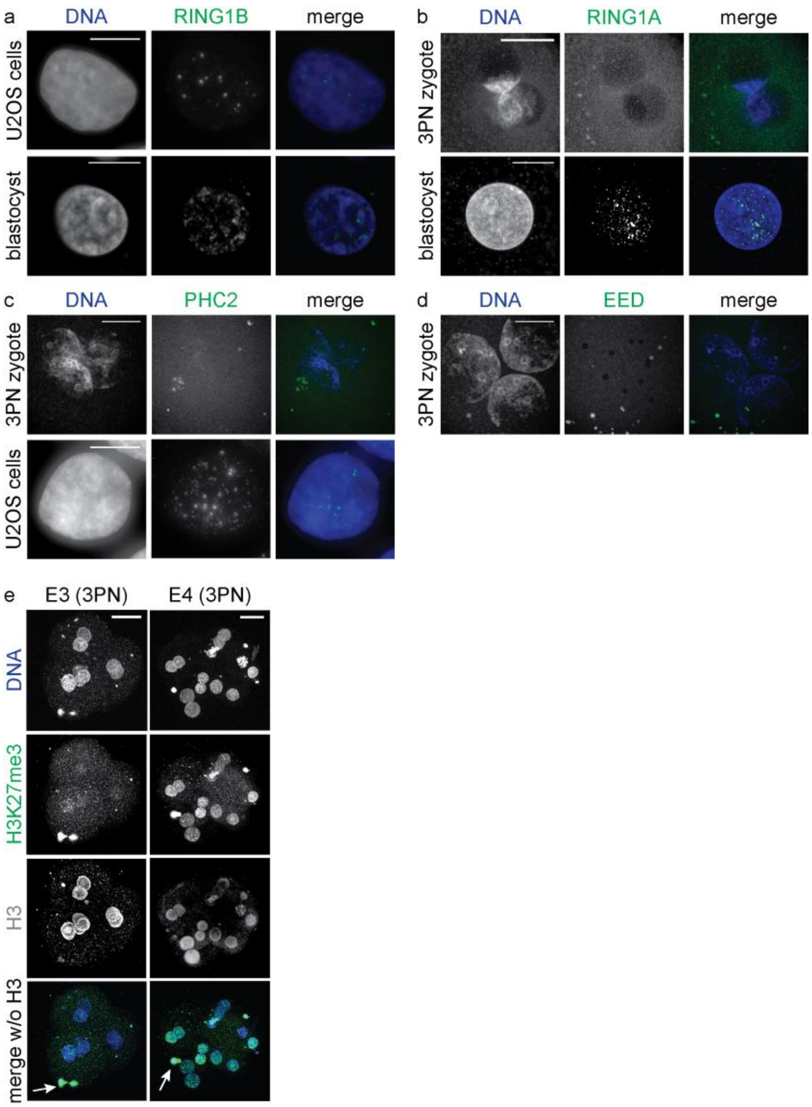

Polycomb Repressive Complex 1 and 2 are not associated with paternal cHC in human embryos until after E3. (**a**) Immunolocalization of PRC1 subunit RING1B (green) in a U2OS cell nucleus and a blastocyst nucleus. Human U2OS cells spontaneously overexpress Polycomb group proteins and serve as a positive antibody control. Shown is a immunofluorescent image of a U2OS cell nucleus containing enrichment of RING1B in typical “polycomb bodies”[25]. Confocal analysis of human blastocytsts fixed on E5 also shows several RING1B foci to be present in the nucleus (n=5). Shown is a representative full projection of Z-sections through a single nucleus. Scale bars, 10μm. (**b**) Immunolocalization of PRC1 subunit RING1A (green) in a 3PN zygote and blastocyst. Representative confocal images of a 3PN zygote fixed 18-20 h post insemination and a blastocyst fixed on E5. Shown are full projections of Z-sections. RING1A is not detected in the zygote (n=5), but several foci can be observed in the blastocyst nucleus (n=5). Scale bars, 30μm (3PN zygote) and 10μm (blastocyst). (**c**) Immunolocalization of PRC1 subunit PHC2 (green) in a 3PN zygote and a U2OS cell nucleus. Representative confocal image of a human 3PN zygote fixed 18-20 h post insemination. Full projections of Z-sections fail to detect PHC2 in the zygote (n=5), but several foci can be

observed in U2OS cells. Scale bar, 30μm (3PN zygote), 10μm (U2OS cell). (**d**) Immunolocalization of PRC2 subunit EED (green) in a 3PN zygote. Representative confocal image of a 3PN zygote fixed 18-20 h post insemination (n=5). Shown is a full projection of Z-sections. EED is not detected at DAPI-dense rings or knobs. Scale bar, 30μm. (**e**) H3K27me3 dynamics in E3 and E4 embryos originating from 3PN embryos. Shown are representative full projections of confocal Z-sections of embryos fixed at indicated developmental stages (n=5 per stage), with immunolocalization of H3K27me3 (green) and histone H3 (white). Results are the same as in embryos from a 2PN origin (compare with Fig. 2e): compared to overall H3 levels, H3K27me3 levels are barely detectable at E3. At E4, high H3K27me3 levels are detected in all nuclei. Arrows indicate the polar body. Scale bars, 30μm.

Supplementary Figure 3

a

GENE	E0	E1	E1.5	E2	E3	E3.5	E4	E5		hESCs
**Controls**										
ZP3	20	20	23	24	24	26	25	27		25
HPRT1	23	24	24	27	27	27	25	24		19
SOX2	31	31	28	26	26	25	23	24		17
OCT4	27	27	28	27	27	25	23	22		15
PRC1										
BMI1	32	34	33	33	30	30	27	26		23
MEL18	29	29	30	30	30	30	29	25		18
RING1A	24	24	25	28	27	26	26	24		18
RING1B	25	26	27	28	27	28	26	25		19
PHC2	26	26	27	29	29	31	29	25		20
CBX2	25	26	26	28	27	30	27	26		20
CBX7	32	32	33	34	34	33	33	31		22
CBX8	25	24	26	28	28	28	27	27		23
PRC2										
EZH1	28	29	29	30	31	32	30	29		21
EZH2	27	27	28	27	27	27	25	24		19
EED	33	36	35	31	31	30	28	26		23

expression	Ct value	color
highly expressed	≤17	
↑	18-20	
	21-23	
	24-26	
	27-29	
below detection threshold	≥30	

b

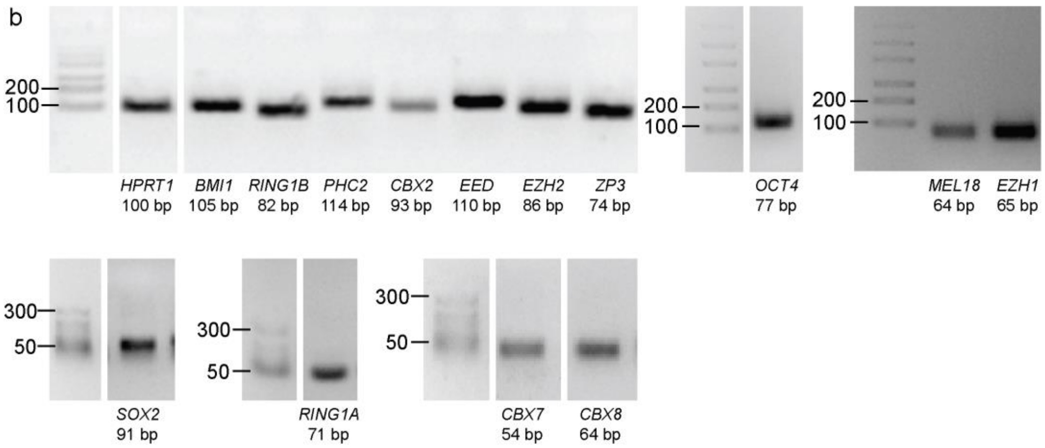

mRNA expression of PRC1/2 subunits in human oocytes and pre-implantation embryos. (**a**) mRNA expression levels of control genes and PRC1/2 subunits were quantified by RT-qPCR in single oocytes and pre-implantation embryos at the following eight developmental stages (Supplementary Fig. 1): E0 (n=7); E1 (n=5); E1.5 (n=5), E2 (n=4), E3 (n=5), E3.5 (n=5), E4 (n=4), and E5 (n=5). Average cycle threshold (Ct) values are given and results are depicted as colours ranging from green to red, indicating expression levels ranging from high (green) to below detection threshold (≥30; red). *ZP3*, expressed only as a maternal transcript, *HPRT1*, expressed stably in somatic cells, and *SOX2* and *OCT4*, transcription factors whose expression is known to increase during pre-implantation development, were used as controls. RNA isolated from human embryonic stem cells (hESCs) is used to verify detection of all investigated mRNAs with the chosen gene expression assays. Expression of the investigated mRNAs followed similar patterns: levels decreased from E0 to E3 and started increasing again around E4. Exceptions were expression of *BMI1* and *EED*, which was not detected until E4, and *CBX7*, which was only detected in hESCs. (**b**) Verification of PCR products on agarose gel. DNA marker sizes are indicated in base pairs. Indicated beneath the gel lanes are the gene and the expected amplicon size in base pairs (bp).

Supplementary Figure 4

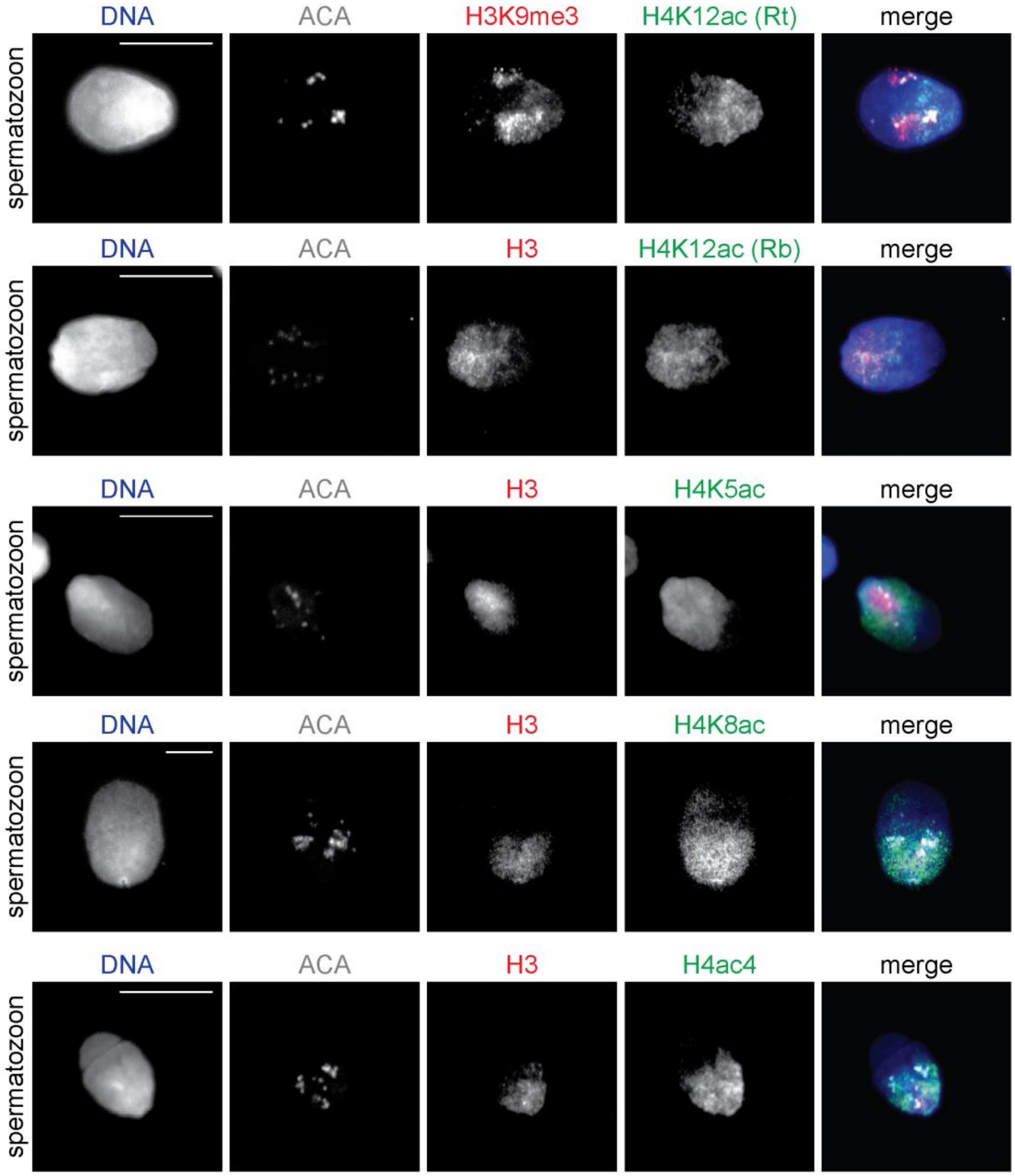

Acetylated forms of Histone 4 are not enriched at pericentric heterochromatin in mature human spermatozoa. Representative full projections of confocal Z-sections through human *in vitro* decondensed spermatozoa (n=100 per experiment). Upper panel: Full set of single channel images corresponding to merged image shown in Fig. 4c, with immunolocalization of H3K9me3 (red) and H4K12ac (monoclonal Rat antibody, green) relative to centromeres (ACA; white). H3K9me3 is enriched in the region surrounding the centromeres, but H4K12ac localizes in a cap-like pattern and is not specifically enriched at pHC. Lower panels: Immunolocalization of chromatin contained in a nucleosomal structure using a histone H3 antibody (green), together with H4K12ac (polyclonal Rabbit antibody), H4K5ac, H4K8ac and a tetra-acetylated form of H4 (red) relative to centromeres (ACA; white). All acetylated forms of H4 were observed to be enriched in a cap-like pattern. Scale bar, 10μm.

Supplementary Figure 5

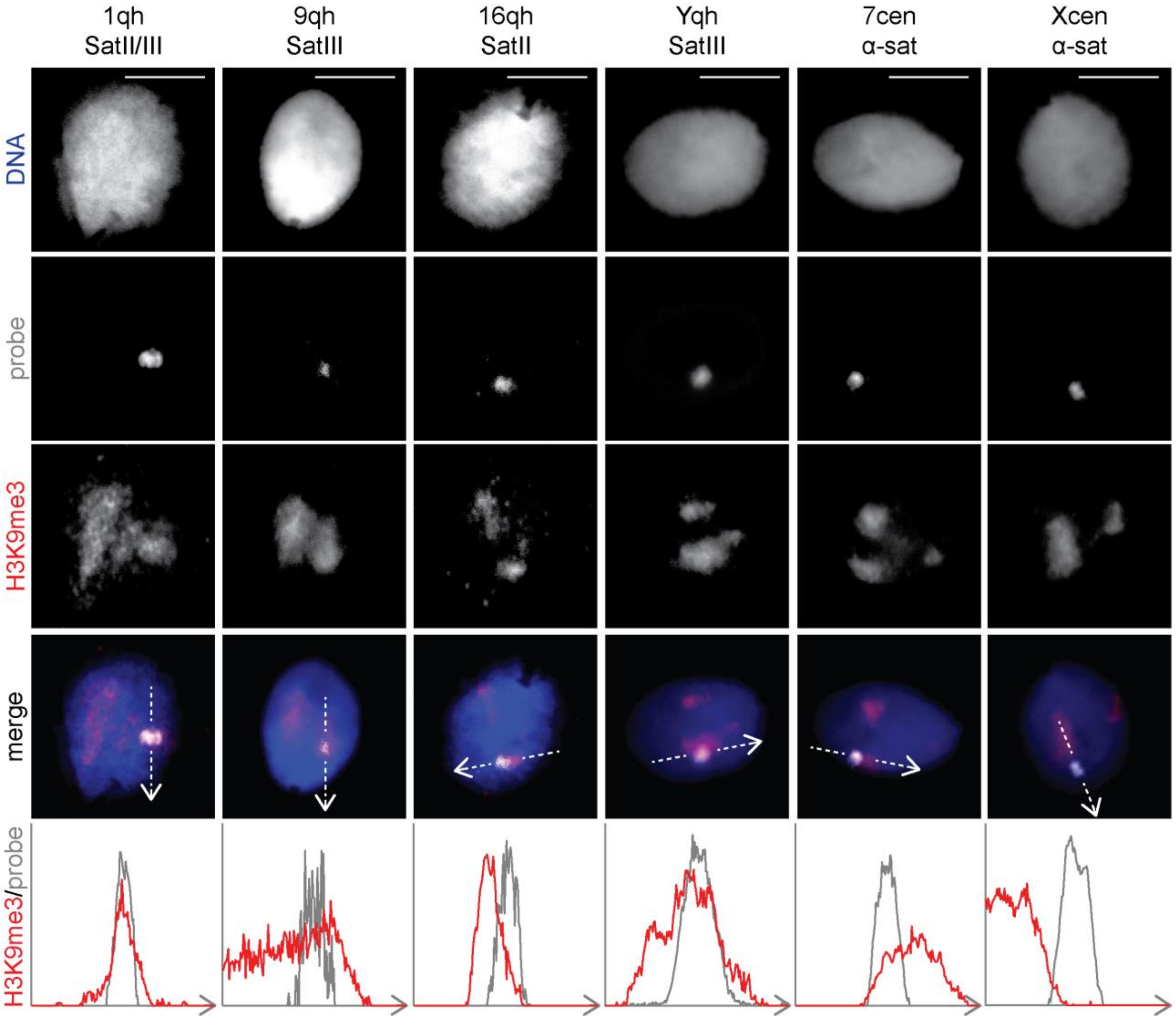

Codetection of H3K9me3 and (peri)centric repeat sequences in *in vitro* decondensed human spermatozoa by immuno-FISH. Full set of single channel images corresponding to merged images shown in Fig. 4d. DNA probes detecting satellite (Sat) DNA II or III repeat sequences at pericentric (chromosomes 1, 9, 16 and Y) and α satellite DNA sequences at centromeric locations (chromosomes 7 and X) are used. Shown are representative single Z-sections for all channels and the merged images. A dotted line was drawn through the probe signal following the length of the H3K9me3 cloud and the distribution of fluorescent intensities along this line were plotted for H3K9me3 (red) and probe signal (grey) in arbitrary units. Scale bar, 5μm.

Supplementary Figure 6

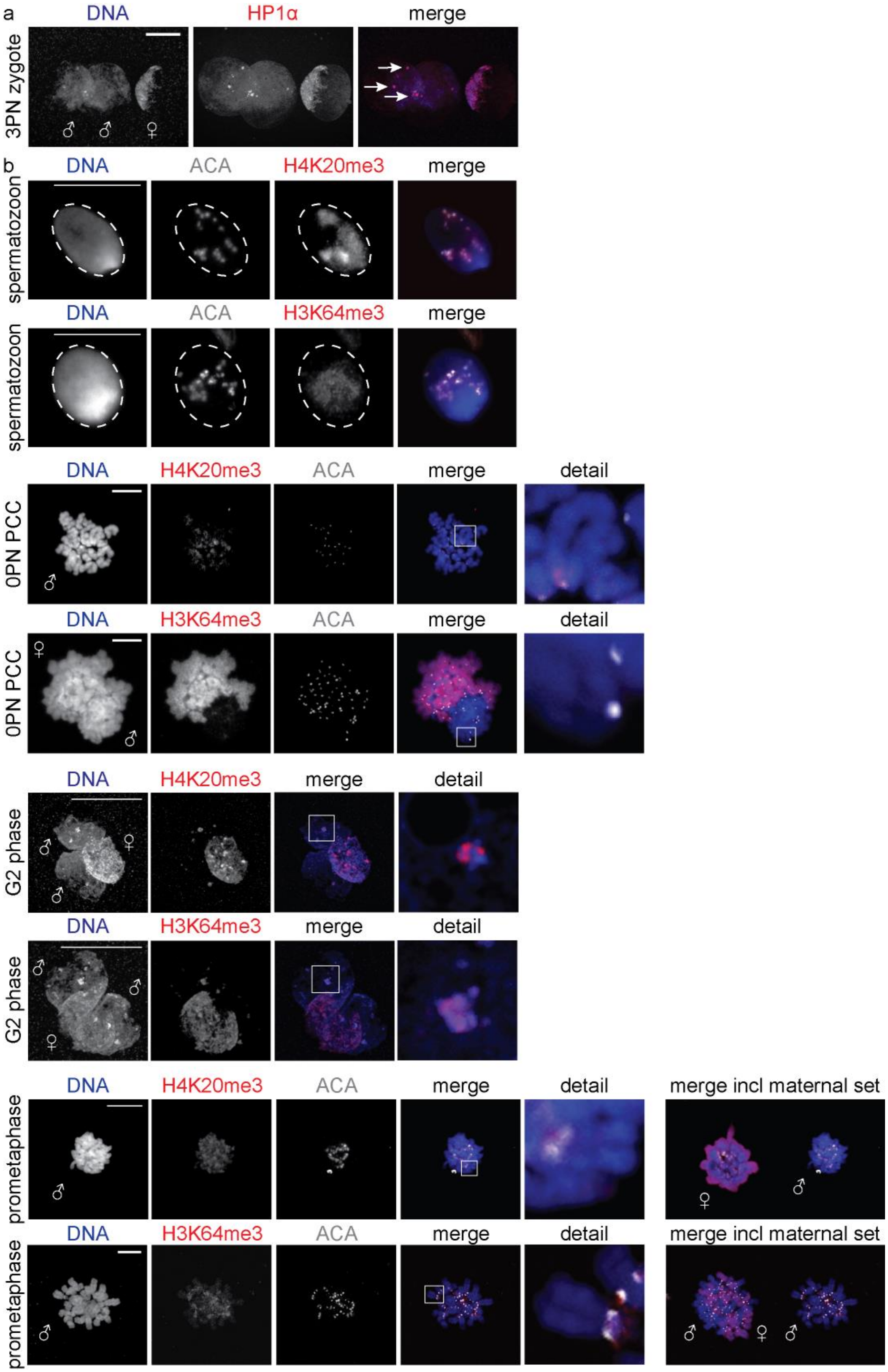

Paternal cHC marked by H3K9me3 also contains HP1α, H4K20me3 and H3K64me3. (**a**) Representative confocal image of a human 3PN zygote fixed 18-20 h post insemination (n=10). Shown is a full projection of Z-sections with immunolocalization of HP1α (red). Paternal (♂) and maternal (♀) pronuclei are indicated. HP1α was detected broadly on the maternal pronucleus and on DAPI-dense knobs on the paternal pronucleus (arrows). Scale bar, 30μm. (**b**) Full set of single channel images corresponding to merged images shown in Fig. 6b/c. Dotted line indicates the sperm nucleus. Paternal (♂) and maternal (♀) chromatin is indicated. Detail shows a magnification of the boxed regions. Scale bar, 10μm (all stages) and 30μm (G2 phase).

Supplementary Figure 7

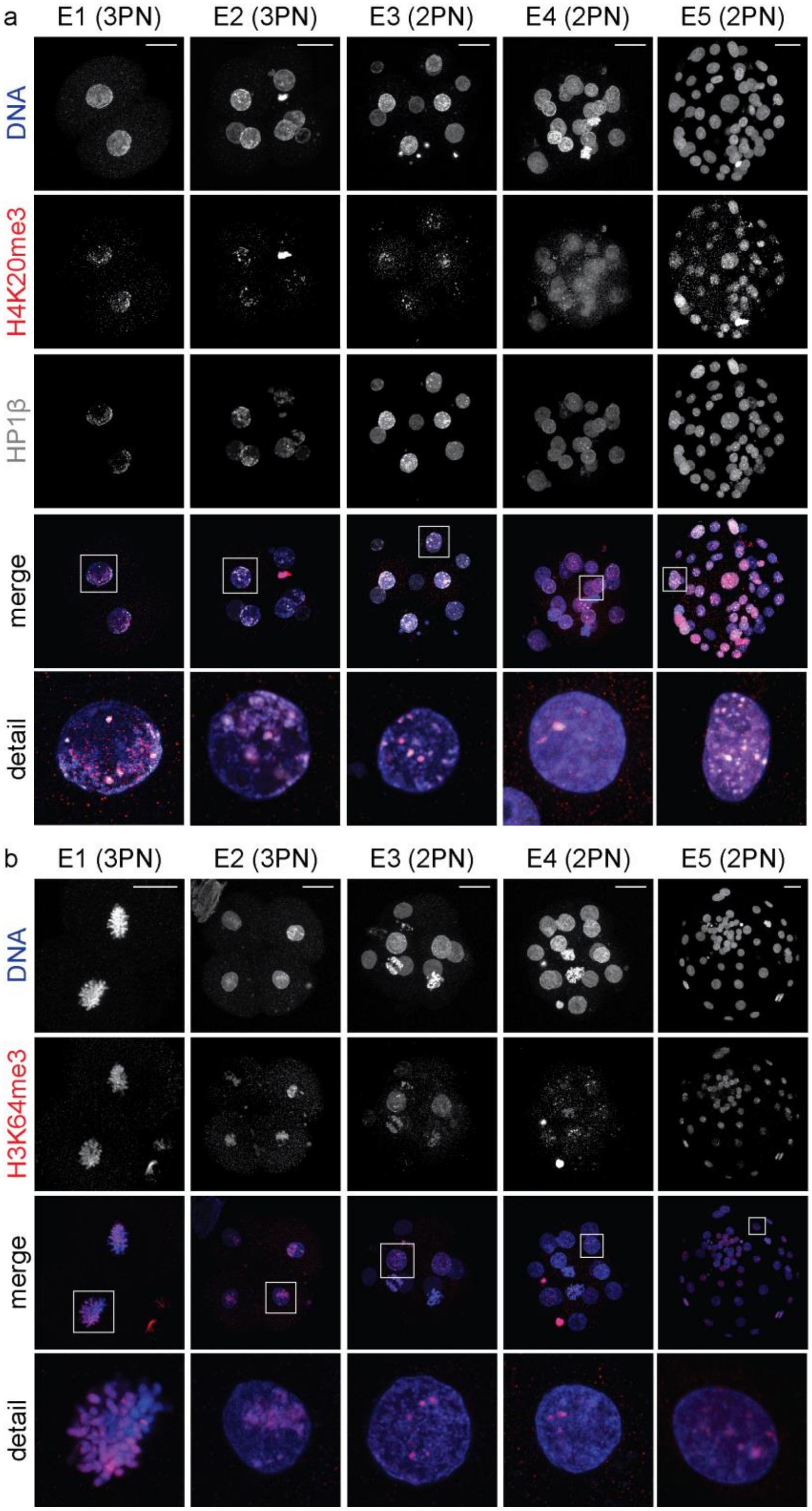

cHC markers HP1β, H4K20me3 and H3K64me3 are maintained during pre-implantation development. (**a, b**) Full set of single channel images corresponding to merged images shown in Fig. 6d/e. Detail shows a magnification of the boxed nucleus. Scale bars, 30μm.
